# Perceived Physical Health and Cognitive Behavioral Therapy vs Supportive Psychotherapy Outcomes in Adults With Late-Life Depression: A Secondary Analysis of a Randomized Clinical Trial

**DOI:** 10.1001/jamanetworkopen.2024.5841

**Published:** 2024-04-15

**Authors:** Forugh S. Dafsari, Bettina Bewernick, Sabine Böhringer, Katharina Domschke, Moritz Elsaesser, Margrit Löbner, Melanie Luppa, Sandra Schmitt, Katja Wingenfeld, Elena Wolf, Nadine Zehender, Martin Hellmich, Wiebke Müller, Michael Wagner, Oliver Peters, Lutz Frölich, Steffi Riedel-Heller, Elisabeth Schramm, Martin Hautzinger, Frank Jessen

**Affiliations:** 1 Department of Psychiatry and Psychotherapy, University of Cologne, Faculty of Medicine, and University Hospital Cologne, Cologne, Germany; 2 Department of Neurodegenerative Diseases and Geriatric Psychiatry, University of Bonn, Bonn, Germany; 3 Department of Psychiatry and Psychotherapy, Medical Center, University of Freiburg, Faculty of Medicine, University of Freiburg, Freiburg, Germany; 4 Institute of Social Medicine, Occupational Health and Public Health, University of Leipzig, Leipzig, Germany; 5 Department of Geriatric Psychiatry, Central Institute of Mental Health, Medical Faculty Mannheim, University of Heidelberg, Mannheim, Germany; 6 Department of Psychiatry and Psychotherapy, Charité–Universitätsmedizin Berlin, Berlin, Germany; 7 Institute of Medical Statistics and Computational Biology, University of Cologne, Faculty of Medicine and University Hospital Cologne, Cologne, Germany; 8 German Center for Neurodegenerative Disease, Bonn, Germany; 9 Department of Clinical Psychology and Psychotherapy, Eberhard Karls University, Tübingen, Germany; 10 Cellular Stress Response in Aging-Associated Diseases Cluster of Excellence, University of Cologne, Faculty of Medicine and University Hospital Cologne, Cologne, Germany

## Abstract

**Importance:**

Physical diseases co-occur with late-life depression (LLD). The influence of physical diseases and the subjective perception of physical health (PPH) on treatment outcome in LLD, however, is not well understood.

**Objective:**

To assess the association of physical diseases and PPH with the outcomes of 2 different types of psychotherapy in LLD.

**Design, Setting, and Participants:**

This post hoc secondary analysis of a multicenter, observer-blinded, controlled, parallel-group randomized clinical trial assessed participants 60 years or older with moderate to severe depression recruited at 7 psychiatric-psychotherapeutic outpatient trial sites in Germany from October 1, 2018, to November 11, 2020. Data analysis was performed from April 1 to October 31, 2023.

**Interventions:**

Patients received LLD-specific cognitive behavioral therapy (LLD-CBT) or supportive unspecific intervention (SUI).

**Main Outcomes and Measures:**

Depression severity, response, and remission were measured during treatment and at 6-month follow-up by the change in the 30-item Geriatric Depression Scale (GDS) score. Physical health and PPH were assessed by the number of physical diseases, Charlson Comorbidity Index (CCI), and the World Health Organization Quality of Life Brief Version physical health subscale.

**Results:**

A total of 251 patients were randomized to LLD-CBT (n = 126) or SUI (n = 125), of whom 229 (mean [SD] age, 70.2 [7.1] years; 151 [66%] female) were included in the intention-to-treat analysis. Patients with low and moderate PPH at baseline had significantly less reduction in the GDS score across both treatment groups than patients with high PPH (estimated marginal mean difference [EMMD], 2.67; 95% CI, 0.37-4.97; *P* = .02 for low PPH and EMMD, 1.82; 95% CI, 0.22-3.42; *P* = .03 for moderate vs high PPH). Higher PPH at baseline was associated with higher likelihood of response (odds ratio [OR], 1.04; 95% CI, 1.00-1.06; *P* = .009) and remission at the end of treatment (OR, 1.04; 95% CI, 1.02-1.08; *P* = .002) and response (OR, 1.05; 95% CI, 1.02-1.08; *P* < .001) and remission at follow-up (OR, 1.06; 95% CI, 1.03-1.10; *P* < .001) across both treatment groups. However, a significant interaction of PPH with treatment group was observed with low PPH at baseline being associated with significantly larger reduction in GDS scores in SUI compared with LLD-CBT at the end of treatment (EMMD, −6.48; 95% CI, −11.31 to −1.64; *P* = .009) and follow-up (EMMD, −6.49; 95% CI, −11.51 to −1.47; *P* = .01). In contrast, patients with high PPH at baseline had a significantly greater reduction in GDS scores in LLD-CBT compared with SUI at all time points (week 5: EMMD, −4.08; 95% CI, −6.49 to −1.67; *P* = .001; end-of-treatment: EMMD, −3.67; 95% CI, −6.72 to −0.61; *P* = .02; and follow-up: EMMD, −3.57; 95% CI, −6.63 to −0.51; *P* = .02). The number of physical diseases or CCI at baseline did not have an effect on the change in GDS score, response, or remission, neither across both groups nor within either group.

**Conclusions and Relevance:**

In this secondary analysis of a randomized clinical trial, subjective PPH was associated with treatment outcome, response, and remission in psychotherapy of LLD. Patients with LLD responded differently to LLD-CBT and SUI, depending on their baseline PPH score. Treatment approaches for patients with LLD should address PPH in personalized interventions.

**Trial Registration:**

ClinicalTrials.gov Identifier: NCT03735576; Deutsches Register Klinischer Studien Identifier: DRKS00013769

## Key Points

**Question:**

How are physical diseases and the subjective perception of physical health (PPH) associated with treatment outcome in psychotherapy of late-life depression (LLD)?

**Findings:**

In this post hoc secondary analysis of a multicenter randomized clinical trial of 229 patients with LLD, patients with high PPH at baseline showed greater reduction in depressive symptoms by LLD-specific cognitive behavioral therapy, whereas patients with low PPH at baseline showed greater reduction by supportive unspecific intervention. Physical diseases did not affect treatment outcome.

**Meaning:**

Subjective PPH is differentially associated with response to LLD-specific cognitive behavioral therapy vs supportive unspecific intervention, which may guide personalized interventions.

## Teaser

This secondary analysis of a randomized clinical trial investigates the association of physical diseases and perception of physical health with psychotherapy treatment outcome, response, and remission in patients in Germany with late-life depression.

## Introduction

Late-life depression (LLD) is often accompanied by physical diseases and mobility restrictions. Previous studies have delineated a link among LLD, physical diseases, and mortality.^1,2^ Physical diseases have been reported to be negatively associated with treatment outcome in LLD.^3,4,5^ A meta-analysis showed a statistically significant pooled effect size in conjunction with a high level of heterogeneity of the included studies.^6^ However, physical diseases were assessed inconsistently in the studies and included objective assessments of somatic diseases and self-ratings of physical health. Importantly, the objective recording of the number of physical diseases and somatic comorbidities needs to be differentiated from the patient’s subjective perception of the somatic illnesses. Furthermore, perceptions of health have been studied as potential factors associated with physical health outcomes and are highly suggestive of mortality in the geriatric population.^7^ Few studies have assessed the association of perceived physical health (PPH) and treatment outcome in LLD.^8^ In a prospective, observational cohort study, poor PPH was associated with longer time to remission in 239 patients with LLD undergoing psychiatric treatment.^7^ In a study in a primary care setting, low PPH was associated with poor remission rates in a care management program for LLD.^9^ Studies on the role of PPH as a variable in psychotherapy treatment outcome in LLD in psychiatric and psychotherapeutic settings are scarce. Most studies are of limited sample size, are single-center studies, or recruited participants through primary care, which limits the generalizability to clinical populations with moderate to severe LLD. Evidence from an open combination treatment trial of nortriptyline and interpersonal psychotherapy for LLD suggested that lower PPH before treatment was associated with lack of response and remission during treatment.^10,11^ In contrast, PPH was not associated with treatment response to cognitive behavioral therapy (CBT) in an exploratory study of 60 patients with LLD.^12^ In summary, the evidence of the association of physical diseases and PPH with psychotherapy treatment outcome in LLD remains inconclusive. Clarifying this association is of particular importance because it might help to understand the factors that are associated with response to psychotherapy in LLD. Identifying factors associated with patient response might assist in treatment decision-making and provide opportunities to improve treatment efficacy in these patients by developing targeted, personalized interventions.

Previous work found that both an LLD-specific CBT (LLD-CBT) and a supportive unspecific intervention (SUI) significantly reduce depressive symptoms in LLD, without evidence of superiority of LLD-CBT.^13^ Here, we present the results of a post hoc secondary analysis of the study^14^ investigating the association of physical diseases and PPH with treatment outcome in both treatment groups. We tested whether high PPH at baseline would be associated with a greater reduction of depression severity and a higher likelihood of response and remission over the course of the treatments. This is the first multicenter study, to our knowledge, to investigate the association of both physical diseases and PPH separately on psychotherapy treatment outcome, response, and remission in LLD in a psychiatric-psychotherapeutic setting.

## Methods

### Study Design

This is a post hoc analysis of the Cognitive Behavioral Therapy for Late-Life Depression (CBTlate) trial.^13^ Details on the study design have been published previously,^14^ and the trial protocol is given in Supplement 1. Patients were recruited from 7 psychiatric university hospitals or psychotherapy university centers in Germany. All participants provided written informed consent before all study procedures. Patients who met eligibility criteria at baseline were randomly assigned (1:1 randomization) to LLD-CBT or SUI. All clinical interviews and outcome assessments were conducted by raters who were blinded to the treatment arm allocation. The study was approved by the University of Cologne Institutional Review Board/Institutional Ethical Committee and by all other local institutional review boards or institutional ethical committees at the sites before initiation of the trial. This study follows the Consolidated Standards of Reporting Trials (CONSORT) reporting guideline for randomized studies (eFigure in Supplement 2).

### Participants

We recruited 251 outpatients aged 60 years or older with LLD who met the diagnostic criteria for moderate to severe major depressive disorder assessed by trained raters using the validated standard clinical Mini-International Neuropsychiatric Interview for *Diagnostic and Statistical Manual of Mental Disorders* (Fifth Edition), version 7.0.2 (see the trial protocol in Supplement 1).^15^ We included participants with a 30-item Geriatric Depression Scale (GDS)^16^ score greater than 10, Quick Inventory of Depressive Symptomatology–Clinician Rating^17^ score greater than 10, and Mini-Mental-Status-Test^18^ score greater than 25. The exclusion criteria are presented in detail in the trial protocol in Supplement 1.

### Outcome Measures

Assessments were conducted at baseline, in week 5, at the end of treatment in week 10 (EOT), and at follow-up 6 months after randomization. Depression severity, response, and remission were measured during treatment and at 6-month follow-up by the change in GDS score. Physical health and PPH were assessed by the number of physical diseases, Charlson Comorbidity Index (CCI), and the World Health Organization Quality of Life Assessment physical health subscale.

### Depressive Symptoms

The primary end point was depressive symptoms, which were measured using the 30-item GDS (range, 0-30, with higher scores indicating more severe symptoms), a widely established, self-report measure of depressive symptoms in older individiuals.^16,19^ The primary outcome was defined as the change in GDS score from baseline to EOT.

### Measures of Physical Health and Comorbidities

As measures of physical health and comorbidities, the number of physical diseases and the CCI were used. The number of physical diseases was identified by the patients’ medical history data and medical records. Physical comorbidities were assessed by the CCI, which is considered the gold standard measure to assess comorbidity in clinical research.^20^

### Perceived Physical Health

The objective measures of physical health were complemented by the assessment of the patient’s PPH. This measure represents the subjective self-perception of physical health in contrast to information obtained from medical history and records. We assessed PPH by the German version of the World Health Organization Quality of Life Brief Version (WHOQOL-BREF) physical health subscale, which is a widely used and standardized questionnaire available in many culturally appropriate language versions that enables the assessment and international comparisons of PPH. Each of the 7 items is rated on a 5-point rating scale by the patient and assesses the subjective perception of physical health (eg, item 15: “How well are you able to get around physically?”). Higher scores indicate better self-rated PPH. The WHOQOL-BREF has good psychometric properties and is a reliable and valid instrument.^21,22^

### Interventions

The interventions included 15 twice-weekly sessions during 8 weeks of manual-based, individual, outpatient treatment in each arm of the trial. The LLD-CBT intervention is a form of CBT with 6 modules tailored to address late-life specific psychological factors.^13,14,23^ In contrast, the active control intervention SUI uses supportive and general but nonspecific interventions. The SUI treatment sessions were unstructured, with content and process determined by the patient.^13^ All therapists were trained in both interventions and delivered either one according to individual randomization. Adherence was ensured by continuous central supervision of all therapists and by recording of all therapy sessions on film with central adherence assessment on randomly selected recordings.^13,14^

### Statistical Analysis

In this post hoc analysis, we applied a mixed model for repeated measures (MMRM) approach to investigate the change in depression severity over time, as measured by the GDS. We investigated whether the variables of interest (PPH score, number of physical diseases, and CCI) at baseline were associated with the trajectory of change in GDS score over time. We performed the analyses with all variables of interest as continuous variables. The change in GDS from baseline to all time points (GDS score at the time point minus baseline GDS score) was evaluated by an MMRM with the fixed effects of baseline GDS score, therapist, treatment group, visit, age, gender, variable of interest, and the interactions of treatment × visit, variable of interest × visit, and variable of interest × treatment (heterogenous first-order autoregressive structured covariance matrix over time) with corresponding marginal means and contrast tests. Variables of interest were included separately in the model. Additionally, the variable PPH was converted from a continuous to a categorical variable for the purpose of comparisons between groups. Previous evidence on WHOQOL-BREF cutoff points in older adults has shown that a score of 60 or greater is sensitive for adequately differentiating older adults with good or satisfactory health from those with worse or unsatisfactory health.^24,25,26^ To analyze the associations with PPH in a more detailed manner, the converted categorical variable had 3 levels: (1) low (≤1 SD below the mean; score of ≤38), (2) moderate (>1 SD below the mean and ≤60; score of 39-60), and (3) high (score of >60). We calculated effect sizes (Cohen *d*) for both treatment groups separately by dividing the difference from the GDS scores at EOT to baseline by the SD of the baseline GDS score.

In addition, we assessed remission (≤10 points on the GDS) and response (all remitted patients and patients with reduction of ≥50% of the baseline GDS score) at EOT and at follow-up. We performed hierarchical logistic regression analyses to identify the contribution of the variables age, gender, GDS score at baseline, number of physical diseases, CCI, and PPH to the dependent variable response and remission at EOT and follow-up, respectively. Age, gender, and GDS score at baseline were entered in step 1. In step 2, the number of physical diseases and CCI were added, and in step 3, the PPH and the interaction of PPH × treatment were included. Linearity was tested using the Box-Tidwell procedure.^27^ Bonferroni correction was applied to all terms in the logistic regression models.^28^ *R*^2^ according to Nagelkerke was calculated as overall effect size measure.^29^ The full analysis set was derived from the intention-to-treat population (ITT; all participants randomized with a valid baseline assessment and at least 1 valid follow-up outcome assessment). Analyses of the participants treated and observed per protocol (PP; all participants without major protocol violations, at least 9 sessions in 1 of the interventions and all outcome assessments) were performed along the same lines as the ITT population. A 2-sided *P* < .05 was considered statistically significant. Data analysis was performed from April 1, 2023, to October 31, 2023. The analyses were performed with SPSS Statistics software, version 28 (IBM Corp).

## Results

### Demographic Data and Baseline Characteristics 

Between October 1, 2018, and November 11, 2020, we randomly assigned 251 patients to LLD-CBT (n = 126) or SUI (n = 125) (eFigure and eTable 1 in Supplement 2). The ITT population consisted of 229 participants, including 115 in LLD-CBT and 114 in SUI. A total of 213 of 251 participants fulfilled the criteria of the PP population, including 105 in the LLD-CBT and 108 in the SUI group. The relevant baseline demographics and clinical characteristics of the sample are described in the Table. The mean (SD) age across both groups was 70.2 (7.1) years. A total of 151 participants (66%) were female and 78 (34%) were male. Three participants (1%) self-identified as Asian, 1 as Hispanic (0.4%), and 225 (98%) as non-Hispanic White. Race was self-reported by study participants, and race categories were defined by investigators based on the US Office of Management and Budget’s revisions to the standards for the classification of federal data on race and ethnicity. A total of 178 participants (78%) were not employed or retired. The participants were all outpatients and resided independently at home alone, with their spouse, or with other family members (siblings or children). At baseline, the mean (SD) GDS score was 20.7 (4.3). The mean (SD) baseline WHOQOL-BREF physical health score was 53.1 (15.1), with a range of 14 to 90. The mean (SD) number of physical diseases was 3.1 (1.9), and the mean (SD) CCI was 3.3 (1.6).

**Table.** Baseline Demographics and Clinical Characteristics of the Intention-to-Treat Sample^a^

Characteristic	LLD-CBT (n = 115)	SUI (n = 114)	Overall (N = 229)
Age, y			
Mean (SD)	69.6 (7.3)	70.7 (6.9)	70.2 (7.1)
Median (IQR) [range]	68 (64-74) [60-92]	70 (65-77) [60-87]	69 (64-75) [60-92]
Gender			
Female	83 (72)	68 (60)	151 (66)
Male	32 (28)	46 (40)	78 (34)
Race and ethnicity			
Asian	2 (2)	1 (1)	3 (1)
Hispanic	1 (1)	0	1 (<1)
Non-Hispanic White	112 (97)	113 (99)	225 (98)
Relationship status			
Single, separated, or widowed	59 (51)	60 (53)	119 (52)
Married or with partner	56 (49)	54 (47)	110 (48)
Living alone	51 (44)	49 (43)	100 (44)
Education level, y			
Mean (SD)	14.7 (2.7)	14.8 (3.7)	14.8 (3.2)
Median (IQR) [range]	15 (12-17) [8-20]	15 (12-17) [8-36]	15 (12-17) [8-36]
Employment			
Currently employed	25 (22)	26 (23)	51 (22)
Unemployed or retired	90 (78)	88 (77)	178 (78)
Baseline number of physical diseases, mean (SD)	3.2 (1.9)	3.1 (1.9)	3.1 (1.9)
Baseline Charlson Comorbidity Index, mean (SD)	3.2 (1.4)	3.4 (1.8)	3.3 (1.6)
Baseline WHOQOL-BREF physical health score (possible range, 0-100)			
Mean (SD)	53.8 (15.8)	52.5 (14.5)	53.1 (15.1)
Median (IQR) [range]	54 (43-64) [14-90]	52 (43-64) [21-82]	54 (43-64) [14-90]
Baseline GDS score (possible range, 0-30), mean (SD)	21.0 (4.3)	20.4 (4.2)	20.7 (4.3)

Abbreviations: GDS, Geriatric Depression Scale; LLD-CBT, late-life depression–specific cognitive behavioral therapy; SUI, supportive unspecific intervention; WHOQOL-BREF, World Health Organization Quality of Life Brief Version.

^a^ Data are presented as number (percentage) of participants unless otherwise indicated.

### Association of Baseline Variables and GDS Scores During Treatment

**Figure 1.**
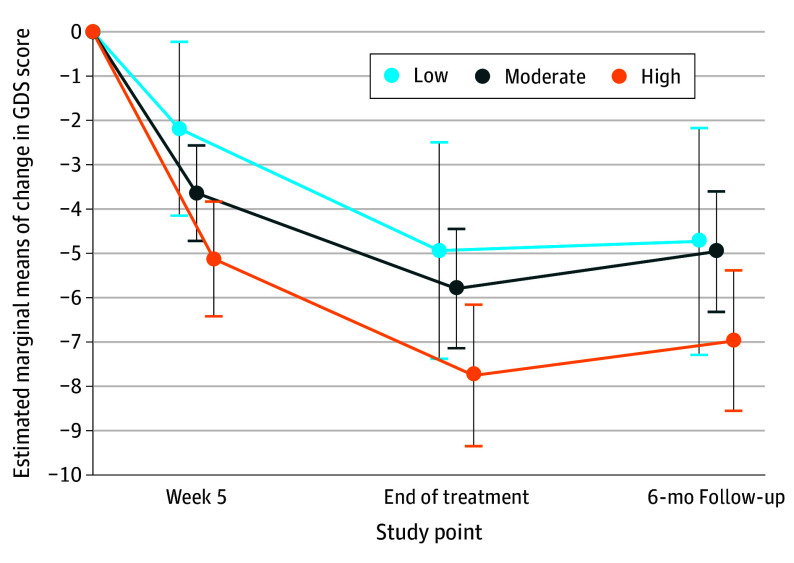
Estimated Marginal Means of the Change in Geriatric Depression Scale (GDS) Score From Baseline to Follow-Up, by Perception of Physical Health at Baseline (Low, Moderate, and High) Assessed With the World Health Organization Quality of Life Brief Version Physical Health Score Error bars indicate the 95% CIs of the estimated marginal means of change in GDS score in the mixed model for repeated measures.

The MMRM showed a significant main effect for age (*F* = 6.53; *P* = .01) and for treatment (*F* = 7.44; *P* = .007). Lower age at baseline was associated with greater reduction of GDS scores than higher age in both treatment groups. There was a significant main effect for the variable PPH at baseline as a continuous variable (*F* = 14.77; *P* < .001), showing that lower scores in PPH at baseline compared with higher scores were associated with less reduction of GDS scores over time across both treatment groups. The main effect for PPH at baseline as a categorical variable based on the converted PPH variable at 3 levels was also significant (*F* = 3.58; *P* = .03). Patients with low and moderate PPH at baseline had significantly less reduction in GDS score than patients with high PPH at baseline (estimated marginal mean difference [EMMD], 2.67; 95% CI, 0.37-4.97; *P* = .02 for low PPH and EMMD, 1.82; 95% CI, 0.22-3.42; *P* = .03 for moderate vs high PPH) (Figure 1). The effect size estimates for the GDS reduction in single-group, pretest-posttest design at EOT were *d* = −1.44 (95% CI, −2.05 to −0.83) for low PPH, *d* = −1.58 (95% CI, −1.90 to −1.25) for moderate PPH, and *d* = −1.91 (95% CI −2.33 to −1.49) for high PPH.

**Figure 2.**
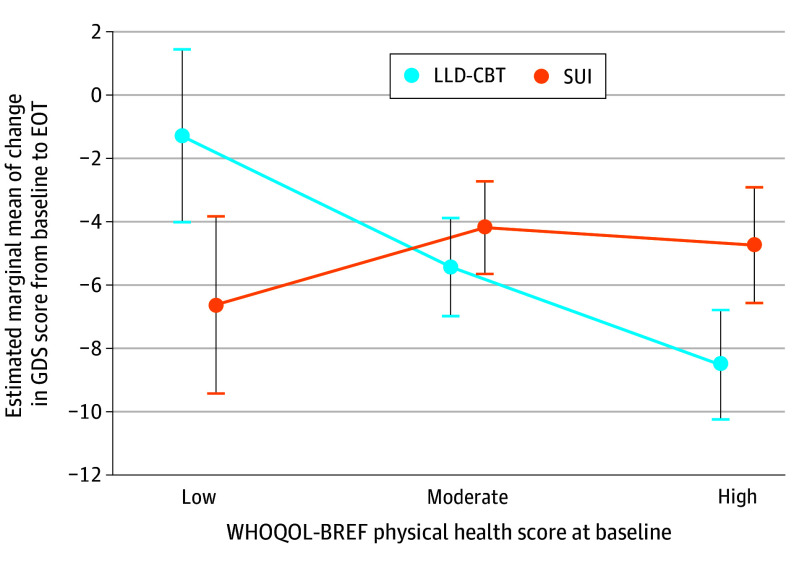
Estimated Marginal Means of the Change in the Geriatric Depression Scale (GDS) Score From Baseline to End of Treatment (EOT) for 3 Groups of Perception of Physical Health at Baseline (Low, Moderate, and High) Assessed With the World Health Organization Quality of Life Brief Version (WHOQOL-BREF) Physical Health Score In patients with low perceived physical health at baseline, the reduction in GDS score from baseline to the end of treatment was significantly larger in the supportive unspecific intervention (SUI) group than in the late-life depression–specific cognitive behavior therapy (LLD-CBT) group. In patients with high perception of physical health at baseline, the reduction in GDS score from baseline to EOT was significantly larger in the LLD-CBT group compared with the SUI group. Error bars indicate the 95% CIs of the estimated marginal means of change in GDS score in the mixed model for repeated measures.

**Figure 3.**
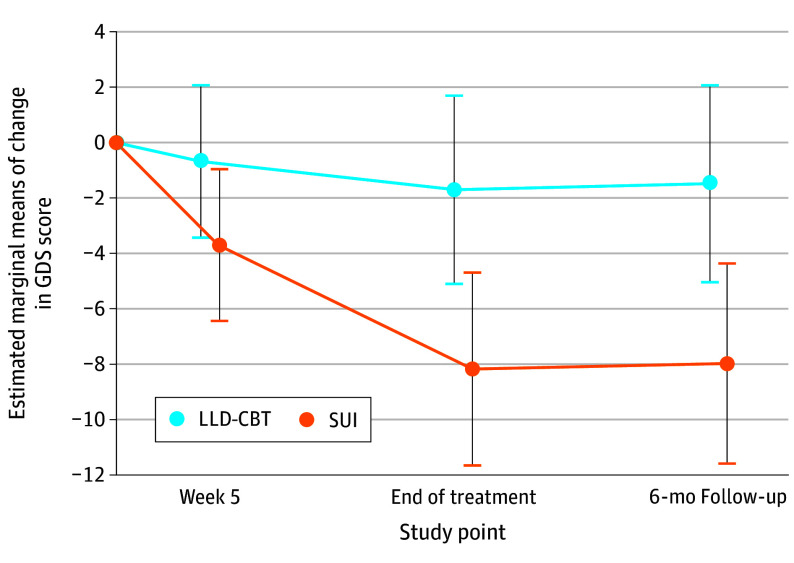
Estimated Marginal Means of the Change in Geriatric Depression Scale (GDS) Score From Baseline to Follow-Up for Patients With Low Perception of Physical Health (World Health Organization Quality of Life Brief Version Physical Health Score ≤38) at Baseline There is a significant difference between the 2 treatment arms in favor of supportive unspecific intervention (SUI) at the end of treatment and follow-up. Error bars indicate the 95% CIs of the estimated marginal means of change in GDS score in the mixed model for repeated measures. LLD-CBT indicates late-life depression–cognitive behavioral therapy.

**Figure 4.**
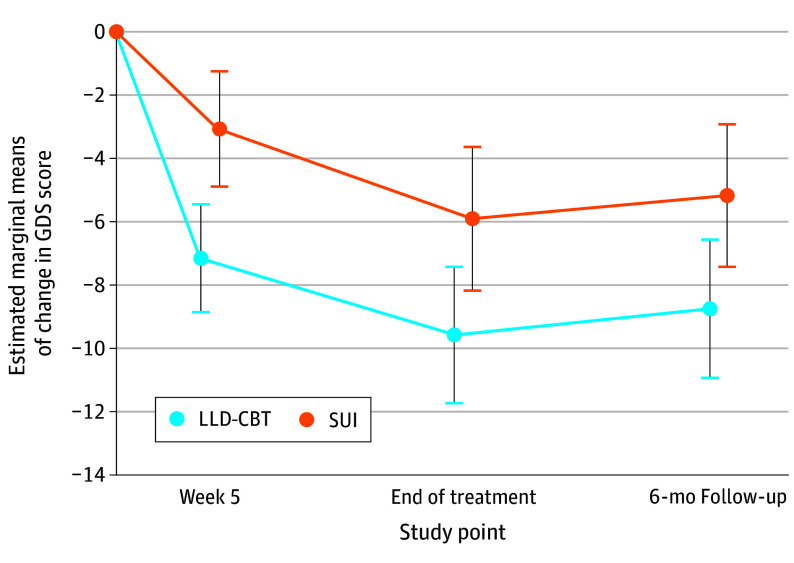
Estimated Marginal Means of the Change in the Geriatric Depression Scale (GDS) Score From Baseline to Follow-Up for Patients With High Perception of Physical Health (World Health Organization Quality of Life Brief Version Physical Health Score >60) at Baseline There is a significant difference between the 2 treatment arms in favor of late-life depression–cognitive behavioral therapy (LLD-CBT) at week 5, end of treatment, and follow-up. Error bars indicate the 95% CIs of the estimated marginal means of change in GDS score in the mixed model for repeated measures. SUI indicates supportive unspecific intervention.

The main effect sizes for sex (*P* = .55), number of physical diseases (*P* = .42), and CCI at baseline (*P* = .69) were not significant. The variable of interest × treatment interaction was significant for PPH at baseline as a continuous (*P* = .001) and categorical variable (*P* = .001) (Figure 2), respectively. In patients with low PPH at baseline, there was a significant between-group difference in the change in GDS scores in favor of SUI (EMMD, −5.33; 95% CI, −9.18 to −1.47; *P* = .007). In these patients, the change in GDS scores was significantly larger in the SUI compared with the LLD-CBT group at EOT (EMMD, −6.48; 95% CI, −11.31 to −1.64; *P* = .009) (Figures 2 and 3) and at follow-up (EMMD, −6.49; 95% CI, −11.51 to −1.47; *P* = .01). The significant effect size difference of the 2 treatment groups at EOT was *d* = −0.74 (95% CI, −1.47 to −0.01), favoring SUI. In the group of patients with high PPH at baseline, there was a significant between-group difference in the change in GDS scores in favor of LLD-CBT at week 5 (EMMD, −4.08; 95% CI, −6.49 to −1.67; *P* = .001), EOT (EMMD, −3.67; 95% CI, −6.72 to −0.61; *P* = .02) (Figure 2), and follow-up (EMMD, −3.57; 95% CI, −6.63 to −0.51; *P* = .02) (Figure 4). The significant effect size difference of the 2 treatment groups at EOT was *d* = −0.67 (95% CI, −1.13 to −0.21), favoring LLD-CBT. In patients with moderate PPH at baseline, there was no significant treatment group difference in the change in GDS scores at any time point. The nonsignificant effect size difference of the treatment groups at EOT was *d* = −0.15 (95% CI, −0.53 to 0.23). The interaction term variable of interest × treatment was not significant for the number of physical diseases or CCI at baseline. All analyses were repeated for the PP population, which confirmed the findings of the ITT population.

### Associations Among Baseline Variables, Response, and Remission

A summary of the 3 steps entered in the hierarchical logistic regression analyses for response and remission at EOT and follow-up are presented in eTables 2 and 3 in Supplement 2. There was no evidence of problems with linearity or multicollinearity. The correlations among the variables included in the models were very low. The correlation between PPH and number of physical diseases was *r* = −0.19, the correlation between PPH and CCI was *r* = −0.10, and the correlation between PPH and GDS score was *r* = −0.28. The correlation was *r* = −0.006 between GDS score at baseline and number of physical diseases and *r* = −0.040 between GDS score at baseline and CCI. The logistic regression for response at EOT was statistically significant for all 3 models (eTable 2 in Supplement 2). There was a significant association between age and response at EOT (odds ratio [OR], 0.93; 95% CI, 0.88-0.98; β [SE] = −0.07 [0.03]; *P* = .006), with higher age being associated with lower likelihood of response at EOT. Furthermore, there was a significant association between PPH at baseline and response at EOT (OR, 1.04; 95% CI, 1.01-1.06; β [SE] = 0.04 [0.01]; *P* = .009), with higher PPH being associated with higher likelihood of response at EOT. The increase in explained variance by the inclusion of PPH in model 3 was 4.9% (Nagelkerke *R*^2^ = 0.14; *P* = .004). The analyses for response at follow-up showed a significant association between PPH at baseline and response at follow-up (OR, 1.05; 95% CI, 1.02-1.08; β [SE] = 0.05 [0.02]; *P* < .001), with higher PPH being associated with higher likelihood of response at follow-up. The response at follow-up was only significant for model 3, resulting in an increase in explained variance of 9.4% (Nagelkerke *R*^2^ = 0.14; *P* = .009). For remission at EOT all models were significant (eTable 2 in Supplement 2). There was a significant association between age and remission at EOT (OR, 0.92; 95% CI, 0.87-0.97; β [SE] = −0.08 [0.03]; *P* = .003), with higher age being associated with lower likelihood of remission at EOT. Furthermore, there was a significant association between PPH at baseline and remission at EOT (OR, 1.04; 95% CI, 1.02-1.08; β [SE] = 0.04 [0.02]; *P* = .002), with higher PPH being associated with higher likelihood of remission at EOT. The increase in variance explained by the inclusion of PPH in model 3 was 7% (Nagelkerke *R*^2^ = 0.20; *P* < .001). The analyses for remission at follow-up showed a significant association between PPH at baseline and remission at follow-up (OR, 1.06; 95% CI, 1.03-1.10; β [SE] = 0.06 [0.02]; *P* < .001), with higher PPH being associated with higher likelihood of remission at follow-up. The analyses for remission at follow-up was only significant for model 3, with an increase in explained variance of 12.1% (Nagelkerke *R*^2^ = 0.16; *P* = .002). Patients with higher PPH at baseline were more likely to respond and to remit at EOT and follow-up in both treatment groups. The interaction term *P* × treatment was not significant for response or remission at any time point.

## Discussion

We investigated the association of physical diseases and PPH with depression severity, response, and remission to LLD-CBT and SUI in patients with LLD. First, our results show that objective measures of physical health (number of physical diseases and somatic comorbidities) were not associated with treatment outcome, response, or remission during psychotherapy in LLD. Patients with LLD benefitted from both treatments regardless of their physical health assessed by medical history, data from medical records, and somatic comorbidities measured with the CCI.

In contrast, the self-rating of physical health was identified as an independent factor associated with the change in depression severity (GDS), response, and remission during LLD-CBT and SUI. High PPH at baseline was associated with greater GDS reduction during treatment and follow-up, whereas low baseline PPH was associated with neither GDS reduction nor response or remission during psychotherapy of LLD.

Second, we found evidence of differential treatment effects of LLD-CBT and SUI on the change in GDS score as a function of PPH at baseline. In patients with high PPH at baseline, there was a significant between-group difference in the change in GDS scores in favor of LLD-CBT compared with SUI at all time points. In contrast, patients with low PPH at baseline showed a larger change in GDS scores from baseline to EOT and follow-up in the SUI compared with the LLD-CBT group.

We conclude that despite the lack of statistically significant differences in the 2 treatments reported from the main analysis of this trial,^13^ there are considerable subgroup interactions. The finding that LLD-CBT had a larger effect size in patients with high PPH might suggest that this group may particularly benefit from a more directive intervention, which requires concrete activities from the patients. Patients with low PPH may be less capable of following a highly structured CBT intervention and may require a less demanding but supportive and accepting approach. Our data demonstrate that the subjective experience of health is more relevant for the treatment response to psychotherapy than actual physical morbidity. Nevertheless, the underlying cause of the differential outcomes remains unclear, and the hypothesized explanation requires subsequent empirical confirmation in additional trials.

As such, our data do not directly support previous studies and a meta-analysis, which reported objective indicators of physical diseases to be negatively associated with treatment outcome in LLD.^3,4,5,6^ This might be explained by the fact that the mentioned meta-analysis^6^ was limited by a high heterogeneity of included studies and consisted of biological, psychosocial, and combined treatment trials (eg, pharmacologic treatments, psychotherapy, and primary care management programs). Additionally, physical disease was assessed inconsistently in the studies and included objective assessments of somatic diseases and self-ratings of physical health. As we showed in our analyses, the objective recording of the number of physical disease and somatic comorbidities should be differentiated from the patient’s subjective perception of the somatic illnesses. Furthermore, in our trial we focused on patients with moderate to severe depression and recruited them from a clinical psychiatric/psychotherapeutic setting. Thus, our sample is distinct from that of other studies because many previous trials^19,30,31,32^ in LLD recruited participants through primary care or as self-referrals. In addition, we recruited a large sample size, which makes our study more robust against type II error. Although a previous study found an association of the PPH with a lack of response and remission in LLD, the authors evaluated an open combination treatment of nortriptyline and interpersonal psychotherapy, which is not equivalent to the comparison of LLD-CBT and SUI in our study.^8,10,11^

### Implications

Our results have several important clinical implications. First, the contrast in response and remission patterns between patients with LLD based on their PPH could provide opportunities to improve treatment efficacy in these patients. Considering individual differences in PPH before treatment as one factor might significantly improve treatment effectiveness for this patient group when selecting a therapeutic modality for patients with LLD. A routine assessment of PPH in clinical practice is still rarely implemented but might be relevant for the selection of an effective treatment. Second, our results imply that physical disease is only associated with treatment response and remission in psychotherapy of LLD when it results in a subjective feeling of reduced physical health and impairment. Because PPH reflects the subjective self-perception of the patient’s physical health, it might be modifiable and targeted in psychotherapeutic interventions. These interventions might include, but are not limited to, changing the patient’s subjective appraisal of physical concerns and developing coping strategies that might help patients better adjust to physical changes at older ages, especially in patients with LLD and low PPH. Thus, innovative psychotherapeutic approaches, such as modular psychotherapy,^33,34,35^ which consider transdiagnostic and individual factors, are needed in patients with LLD to integrate the change of PPH into the therapeutic process. Future studies should investigate the effectiveness of these treatment approaches in patients with LLD and take PPH as an individual baseline variable into account.

### Limitations

The current findings should be considered in the context of several limitations. First, our study did not include a less intense control condition or waiting list to test the effect directly against the untreated natural course of the disorder. Second, this is a post hoc analysis of the CBTlate trial, and the results from these secondary analyses are exploratory. As such, they are hypothesis-generating but not confirmatory. Third, the cutoff scores used for the 3 categories of the WHOQOL-BREF physical health score have not previously been specifically used in a study sample of patients with LLD and have to be evaluated in additional studies. The categorical PPH variable was based on the converted variable at 3 levels and not on an often-used dichotomous cutoff. Fourth, we cannot exclude conclusively that our results may be influenced by variables not assessed in our trial. However, we performed additional moderator analyses on the effects of age, gender, baseline severity of depression, personality traits, cognition, antidepressant medication, socioeconomic status, years of education, and relationship status at baseline on the association of PPH and treatment outcome, which showed no significant results. Thus, additional randomized clinical trials are needed to confirm these results and evaluate the association between PPH and treatment outcome, response, and remission in different psychotherapies for LLD.

## Conclusions

To the best of our knowledge, this secondary analysis of a clinical trial is the first study to demonstrate differential treatment outcomes of LLD-CBT and SUI in LLD as a function of PPH. Our results suggest that response to psychotherapy in LLD is heterogeneous and patients with varying degrees of PPH respond differently to psychotherapeutic interventions. Future efforts should address the improvement of PPH as part of a targeted, personalized intervention to increase rates of remission and response to psychotherapeutic interventions.

## Notes

Supplement 1.
Trial Protocol and Statistical Analysis Plan

Supplement 2. **eFigure.** CONSORT Diagram **eTable 1.** Comparison of Outcome Measures Between the LLD-CBT and SUI Group (ITT Population) Over the Course of the Treatment **eTable 2.** Model Summary of the Hierarchical Logistic Regression Analyses of Response and Remission at the End-of-Treatment and Follow-Up on the Variables of Interest **eTable 3.** Results of the Hierarchical Logistic Regression Analyses of Response and Remission at the End-of-Treatment and Follow-Up for All Variables of Interest

Supplement 3.
Data Sharing Statement

## References

[1] Gleason OC, Pierce AM, Walker AE, Warnock JK. The two-way relationship between medical illness and late-life depression. Psychiatr Clin North Am. 2013;36(4):533-544. doi:10.1016/j.psc.2013.08.003 24229655

[2] Alexopoulos GS. Mechanisms and treatment of late-life depression. Transl Psychiatry. 2019;9(1):188. doi:10.1038/s41398-019-0514-6 31383842 PMC6683149

[3] Alexopoulos GS, Kiosses DN, Sirey JA, . Untangling therapeutic ingredients of a personalized intervention for patients with depression and severe COPD. Am J Geriatr Psychiatry. 2014;22(11):1316-1324. doi:10.1016/j.jagp.2013.05.006 23954038 PMC3923856

[4] Azar AR, Chopra MP, Cho LY, Coakley E, Rudolph JL. Remission in major depression: results from a geriatric primary care population. Int J Geriatr Psychiatry. 2011;26(1):48-55. doi:10.1002/gps.2485 21157850 PMC3049170

[5] Chan MF, Leong KSP, Heng BL, . Reducing depression among community-dwelling older adults using life-story review: a pilot study. Geriatr Nurs. 2014;35(2):105-110. doi:10.1016/j.gerinurse.2013.10.011 24246689

[6] Tunvirachaisakul C, Gould RL, Coulson MC, . Predictors of treatment outcome in depression in later life: a systematic review and meta-analysis. J Affect Disord. 2018;227:164-182. doi:10.1016/j.jad.2017.10.008 29100149

[7] Bosworth HB, McQuoid DR, George LK, Steffens DC. Time-to-remission from geriatric depression: psychosocial and clinical factors. Am J Geriatr Psychiatry. 2002;10(5):551-559. doi:10.1097/00019442-200209000-00008 12213689

[8] Kiosses DN, Leon AC, Areán PA. Psychosocial interventions for late-life major depression: evidence-based treatments, predictors of treatment outcomes, and moderators of treatment effects. Psychiatr Clin North Am. 2011;34(2):377-401, viii. doi:10.1016/j.psc.2011.03.001 21536164 PMC3099466

[9] Alexopoulos GS, Katz IR, Bruce ML, ; PROSPECT Group. Remission in depressed geriatric primary care patients: a report from the PROSPECT study. Am J Psychiatry. 2005;162(4):718-724. doi:10.1176/appi.ajp.162.4.718 15800144 PMC2803683

[10] Miller MD, Schulz R, Paradis C, . Changes in perceived health status of depressed elderly patients treated until remission. Am J Psychiatry. 1996;153(10):1350-1352. doi:10.1176/ajp.153.10.1350 8831449

[11] Lenze EJ, Miller MD, Dew MA, . Subjective health measures and acute treatment outcomes in geriatric depression. Int J Geriatr Psychiatry. 2001;16(12):1149-1155. doi:10.1002/gps.503 11748774

[12] Marquett RM, Thompson LW, Reiser RP, . Psychosocial predictors of treatment response to cognitive-behavior therapy for late-life depression: an exploratory study. Aging Ment Health. 2013;17(7):830-838. doi:10.1080/13607863.2013.791661 23631698 PMC3755101

[13] Dafsari FS, Bewernick B, Böhringer S, . Cognitive Behavioral Therapy for Late-Life Depression (CBTlate): results of a multicenter, randomized, observer-blinded, controlled trial. Psychother Psychosom. 2023;92(3):180-192. doi:10.1159/000529445 37004508

[14] Dafsari FS, Bewernick B, Biewer M, . Cognitive behavioural therapy for the treatment of late life depression: study protocol of a multicentre, randomized, observer-blinded, controlled trial (CBTlate). BMC Psychiatry. 2019;19(1):423. doi:10.1186/s12888-019-2412-0 31881995 PMC6935201

[15] Sheehan DV, Lecrubier Y, Sheehan KH, . The Mini-International Neuropsychiatric Interview (M.I.N.I.): the development and validation of a structured diagnostic psychiatric interview for DSM-IV and ICD-10. J Clin Psychiatry. 1998;59(suppl 20):22-33. 9881538

[16] Yesavage JA, Brink TL, Rose TL, . Development and validation of a geriatric depression screening scale: a preliminary report. J Psychiatr Res. 1982;17(1):37-49. doi:10.1016/0022-3956(82)90033-4 7183759

[17] Rush AJ, Trivedi MH, Ibrahim HM, . The 16-Item Quick Inventory of Depressive Symptomatology (QIDS), clinician rating (QIDS-C), and self-report (QIDS-SR): a psychometric evaluation in patients with chronic major depression. Biol Psychiatry. 2003;54(5):573-583. doi:10.1016/S0006-3223(02)01866-8 12946886

[18] Kessler J, Folstein SE, Denzler P. MMST. Mini-Mental-Status-Test. Deutschsprachige Fassung. Beltz; 1990.

[19] Cuijpers P, Karyotaki E, Pot AM, Park M, Reynolds CF III. Managing depression in older age: psychological interventions. Maturitas. 2014;79(2):160-169. doi:10.1016/j.maturitas.2014.05.027 24973043 PMC4537161

[20] Charlson ME, Carrozzino D, Guidi J, Patierno C. Charlson Comorbidity Index: a critical review of clinimetric properties. Psychother Psychosom. 2022;91(1):8-35. doi:10.1159/000521288 34991091

[21] Conrad I, Matschinger I, Kilian R, Riedel-Heller SG. WHOQOL-OLD und WHOQOL-BREF—Handbuch für die deutschsprachigen Versionen der WHO-Instrumente zur Erfassung von Lebensqualität im Alter. Hogrefe Verlag; 2016.

[22] The WHOQOL Group. Development of the World Health Organization WHOQOL-BREF quality of life assessment. Psychol Med. 1998;28(3):551-558. doi:10.1017/S0033291798006667 9626712

[23] Hautzinger M. Depression im Alter. Psychotherapeutische Behandlung für das Einzel- und Gruppensetting. 2nd ed. Beltz; 2016.

[24] Silva SM, Santana ANC, Silva NNBD, Novaes MRCG. VES-13 and WHOQOL-bref cutoff points to detect quality of life in older adults in primary health care. Rev Saude Publica. 2019;53:26. doi:10.11606/S1518-8787.2019053000802 30942268 PMC6474744

[25] Henchoz Y, Botrugno F, Cornaz S, Büla C, Charef S, Santos-Eggimann B; Research Group on the quality of life of older people in cantons of Vaud and Geneva. Determinants of quality of life in community-dwelling older adults: comparing three cut-offs on the excellent-to-poor spectrum. Qual Life Res. 2017;26(2):283-289. doi:10.1007/s11136-016-1394-3 27558783

[26] dos Santos Tavares DM, Fernandes Bolina A, Aparecida Dias F, dos Santos Ferreira PC, José Haas V. Quality of life of elderly. Comparison between urban and rural areas. Invest Educ Enferm. 2014;32(3):401-413. doi:10.17533/udea.iee.v32n3a05 25504406

[27] Box GEP, Tidwell PW. Transformation of the independent variables. Technometrics. 1962;4(4):531-550. doi:10.1080/00401706.1962.10490038

[28] Tabachnick BG, Fidell LS. Using Multivariate Statistics. 7th ed. Pearson Education; 2018.

[29] Nagelkerke NJD. A note on a general definition of the coefficient of determination. Biometrika. 1991;78(3):691-692. doi:10.1093/biomet/78.3.691

[30] Cuijpers P, van Straten A, Smit F. Psychological treatment of late-life depression: a meta-analysis of randomized controlled trials. Int J Geriatr Psychiatry. 2006;21(12):1139-1149. doi:10.1002/gps.1620 16955421

[31] Huang AX, Delucchi K, Dunn LB, Nelson JC. A systematic review and meta-analysis of psychotherapy for late-life depression. Am J Geriatr Psychiatry. 2015;23(3):261-273. doi:10.1016/j.jagp.2014.04.003 24856580

[32] Chen YJ, Li XX, Pan B, . Non-pharmacological interventions for older adults with depressive symptoms: a network meta-analysis of 35 randomized controlled trials. Aging Ment Health. 2021;25(5):773-786. doi:10.1080/13607863.2019.1704219 31880174

[33] Herpertz SC, Schramm E. Modulare Psychotherapie: Ein Mechanismus-basiertes, personalisiertes Vorgehen. Schattauer; 2022.

[34] Dalgleish T, Black M, Johnston D, Bevan A. Transdiagnostic approaches to mental health problems: current status and future directions. J Consult Clin Psychol. 2020;88(3):179-195. doi:10.1037/ccp0000482 32068421 PMC7027356

[35] Elsaesser M, Herpertz S, Piosczyk H, Jenkner C, Hautzinger M, Schramm E. Modular-based psychotherapy (MoBa) versus cognitive-behavioural therapy (CBT) for patients with depression, comorbidities and a history of childhood maltreatment: study protocol for a randomised controlled feasibility trial. BMJ Open. 2022;12(7):e057672. doi:10.1136/bmjopen-2021-057672 35820739 PMC9277372

