# Supplementary material for: Perceived Physical Health and Cognitive Behavioral Therapy vs Supportive Psychotherapy Outcomes in Adults With Late-Life Depression: A Secondary Analysis of a Randomized Clinical Trial
Source: JAMA Netw Open. 2024 Apr 15;7(4):e245841. doi: 10.1001/jamanetworkopen.2024.5841 (PMC11019392; doi:10.1001/jamanetworkopen.2024.5841)
Supplement: Supplement 1. — Trial Protocol and Statistical Analysis Plan [file jamanetwopen-e245841-s001.pdf]

# Perceived physical health and cognitive behavioral therapy vs supportive psychotherapy outcomes in adults with late-life depression: a secondary analysis of a randomized clinical trial

Forugh S. Dafsari, MD, Bettina Bewernick, PhD, Sabine Böhringer, MSc, Katharina Domschke, MD, PhD, Moritz Elsaesser, MSc, Margrit Löbner, PhD, Melanie Luppa, PhD, Sandra Schmitt, MSc, Katja Wingenfeld, PhD, Elena Wolf, MSc, Nadine Zehender, MSc, Martin Hellmich, PhD, Wiebke Müller, MSc, Michael Wagner, PhD, Oliver Peters, MD, Lutz Frölich, MD, Steffi Riedel-Heller, MD, Elisabeth Schramm, PhD, Martin Hautzinger, PhD, Frank Jessen, MD

# Supplement 1

# Table of contents


**1 STUDY PROTOCOL** ... **4**

1.1 PROTOCOL SYNOPSIS ... 4

1.2 FINANCING OF TRIAL ... 7

1.3 TABLE OF CONTENTS (REMOVED FROM HERE) ... 8

*1.3.1 List of in-text tables ... 8*

*1.3.2 List of in-text figures ... 8*

1.4 LIST OF ABBREVIATIONS ... 8

1.5 ETHICS ... 9

*1.5.1 Independent Ethics Committee ... 9*

*1.5.2 Ethical conduct of the trial ... 9*

*1.5.3 Legislation and guidelines used for preparation ... 9*

*1.5.4 Notification of the authorities, approval and registration ... 9*

*1.5.5 Subject information and consent ... 9*

*1.5.6 Insurance ... 10*

*1.5.7 Subject data and data protection ... 10*

*1.5.8 Point of contact ... 10*

1.6 TRIAL ADMINISTRATIVE STRUCTURE ... 10

1.7 SCIENTIFIC BACKGROUND AND RATIONALE ... 11

*1.7.1 Benefit/risk analysis ... 12*

1.8 STUDY OBJECTIVES AND ENDPOINT CRITERIA ... 13

*1.8.1 Objectives ... 13*

1.8.1.1 Primary Study Objectives ... 13

1.8.1.2 Secondary Study Objectives ... 13

*1.8.2 Endpoint criteria ... 13*

1.8.2.1 Primary endpoint criteria ... 13

1.8.2.2 Secondary endpoint criteria ... 13

1.9 TRIAL DESIGN AND METHODOLOGY ... 14

*1.9.1 Trial design ... 14*

*1.9.2 Discussion of trial design ... 14*

*1.9.3 Blinding ... 15*

*1.9.4 Time schedule ... 15*

*1.9.5 End of trial ... 15*

1.10 TRIAL POPULATION ... 15

*1.10.1 Definitions ... 15*

*1.10.2 Selection of trial population ... 16*

1.10.2.1 Recruitment procedure ... 16

1.10.2.2 Screening log ... 16

1.10.2.3 Inclusion criteria ... 16

1.10.2.4 Exclusion criteria ... 16

*1.10.3 Removal of subjects from the trial ... 17*

1.10.3.1 Notification of discontinuations ... 18

*1.10.4 Trial completion ... 18*

1.10.4.1 Subject definition and subject completion ... 18

1.10.4.2 Procedures for handling of replacements, withdrawals or drop-outs ... 18

1.10.4.3 Premature termination of the trial ... 18

1.11 TRIAL PROCEDURES ... 19

*1.11.1 Flow chart ... 19*

*1.11.2 Trial schedule ... 20*

*1.11.3 Course of the trial ... 22*

1.11.3.1 Information of subjects and informed consent ... 22

1.11.3.2 Enrollment and enrollment visit ........ 22
1.11.3.3 Treatment Phase ........ 23
**1.11.3.3.1 LLD-CBT** ........ 24
**1.11.3.3.2 LLD-SUI** ........ 24
**1.11.3.3.3 Allocation to Interventions** ........ 25
1.11.3.4 Post-treatment phase ........ 25
1.11.3.5 Final examination ........ 25
1.11.3.6 Examination hierarchy and time windows ........ 26
*1.11.4 Trial conditions and restrictions ........ 26*
1.11.4.1 Medical surveillance ........ 26
1.11.4.2 Precaution and emergencies ........ 27
1.11.4.3 General restrictions ........ 27
1.12 TRIAL VARIABLES ........ 27
*1.12.1 Demography ........ 27*
*1.12.2 Adverse events ........ 27*
1.12.2.1 Definition of adverse events ........ 27
1.12.2.2 Definition of serious adverse events ........ 28
1.12.2.3 Definition of intensity of adverse events ........ 28
1.12.2.4 Definition of serious adverse event and adverse event causality ........ 28
1.12.2.5 Definition of measures in case of serious adverse event and adverse event ........ 29
1.12.2.6 Definition of serious adverse event and adverse event outcome ........ 29
1.12.2.7 Suicidality ........ 29
1.12.2.8 Adverse event recording and reporting ........ 30
1.12.2.9 Annual safety report ........ 30
1.13 DOCUMENTATION AND ARCHIVING OF TRIAL DATA ........ 30
*1.13.1 Data Management ........ 30*
*1.13.2 Electronic Case Report Form (eCRF) ........ 31*
*1.13.3 Source data ........ 31*
1.13.3.1 Coding ........ 31
*1.13.4 Data archiving ........ 31*
1.14 STATISTICAL METHODS ........ 31
*1.14.1 Determination of sample size ........ 31*
*1.14.2 Randomization ........ 32*
*1.14.3 Analysis of the trial ........ 32*
1.14.3.1 Analysis of demographic data and other data of interest ........ 33
1.14.3.2 Safety analysis ........ 33
1.15 DATA QUALITY ASSURANCE ........ 33
*1.15.1 Monitoring ........ 33*
*1.15.2 Quality system, audit and inspection ........ 33*
1.15.2.1 Quality system ........ 33
1.15.2.2 Audit/Inspections ........ 33
1.16 GENERAL CONDITIONS AND AGREEMENTS ........ 33
*1.16.1 Amendment of protocol ........ 33*
*1.16.2 Publication policy ........ 34*
*1.16.3 Interim reports ........ 34*
*1.16.4 Final report ........ 35*
*1.16.5 Confidentiality of trial results ........ 35*
1.17 REFERENCES ........ 35
1.18 APPENDIX ........ 38
*1.18.1 Planned participating trial sites ........ 38*

**2 STATISTICAL ANALYSIS PLAN ........ 38**

2.1 BACKGROUND ........ 38
*2.1.1 Trial objective ........ 38*

*2.1.2 Trial design* ... *38*
*2.1.3 Treatments* ... *38*
*2.1.4 Blinding* ... *39*
*2.1.5 Randomisation* ... *39*
*2.1.6 Timing of Analyses* ... *40*
*2.1.7 Sample size* ... *40*
2.2 ANALYSIS POPULATIONS ... 40
*2.2.1 Definitions* ... *40*
*2.2.2 Major protocol violations / Withdrawals* ... *40*
2.3 TRIAL CENTRES ... 41
2.4 ANALYSIS VARIABLES ... 41
*2.4.1 Demography and baseline characteristics* ... *41*
*2.4.2 Primary variable* ... *42*
*2.4.3 Secondary variables* ... *42*
2.4.3.1 Safety/Tolerability ... 42
2.4.3.2 Quality of life ... 42
2.5 HANDLING OF MISSING VALUES AND OUTLIERS ... 42
*2.5.1 Missing values* ... *42*
*2.5.2 Outliers* ... *42*
2.6 STATISTICAL ANALYSES / METHODS ... 43
*2.6.1 Patient / Subject Disposition* ... *43*
*2.6.2 Demography and baseline characteristics* ... *43*
*2.6.3 Primary analysis* ... *43*
*2.6.4 Secondary analyses* ... *43*
2.6.4.1 Safety/Tolerability ... 44
**2.6.4.1.1 Adverse events** ... 44
**2.6.4.1.2 Laboratory parameters** ... 44
2.6.4.2 Quality of life ... 44
*2.6.5 Planned subgroup analyses* ... *44*
*2.6.6 Interim analyses* ... *45*
2.7 DATA PROBLEMS ... 45
2.8 SOFTWARE ... 45
*2.8.1 List of abbreviations* ... *45*

# 1 Study Protocol

**Cognitive behavioural therapy for the treatment of late life depression – a multicentre, randomized, observer-blinded, controlled trial (CBTlate)**

| | |
|---|---|
| **Sponsor** | **University of Cologne**<br>Albertus-Magnus-Platz<br>50923 Cologne<br>Germany |
| **Sponsor's representative and Lead Principal Investigator** | **Prof. Dr. med. Frank Jessen**<br>Department of Psychiatry and Psychotherapy<br>University of Cologne<br>Medical Faculty<br>Kerpener Strasse 62<br>50937 Cologne<br>Germany |
| **Protocol Number** | **UzK-Nr.: Uni-Koeln- 3236** |
| **Version** | **V01_0** |
| **Date of Protocol** | **20.03.2018** |

## 1.1 PROTOCOL SYNOPSIS

| | |
|---|---|
| **Sponsor** | University of Cologne<br>Albertus-Magnus-Platz<br>50923 Cologne |
| **Trial Identification Code** | CBTlate |
| **Sponsor's representative and Lead Principal Investigator** | Prof. Dr. med. Frank Jessen<br>Department of Psychiatry and Psychotherapy<br>University of Cologne<br>Medical Faculty<br>Kerpener Str. 62<br>50937 Köln<br>Germany<br>Tel.: +49-221-478- 4010<br>Fax: +49-221-478-5593<br>E-mail: frank.jessen@uk-koeln.de |
| **Co-Lead Principal Investigator** | Prof. Dr. phil. Martin Hautzinger<br>Department of Psychology |

| | |
|---|---|
| | Clinical Psychology and Psychotherapy<br>Eberhard-Karls-University<br>Schleichstrasse 4<br>72076 Tuebingen<br>Germany<br>Tel. +49-7071-2977301<br>Fax: +49-7071-295219<br>Email: hautzinger@uni-tuebingen.de |
| **Protocol Number** | **UzK-Nr.: Uni-Koeln- 3236** |
| **Title** | Cognitive behavioural therapy for the treatment of late life depression – a multicentre, randomized, observer-blinded, controlled trial (CBTlate) |
| **Trial Design** | Randomized, multi-center, single blind (observer-blinded), active-controlled, parallel group trial (therapeutic confirmatory) in 248 patients with late-life depression of both genders. |
| **Condition** | Late life depression (age ≥ 60 years) |
| **Objective** | To test the hypothesis that a 15-session individually-delivered cognitive behavioural therapy (CBT) specific for late life depression (LLD) is of superior efficacy in reducing symptoms of depression in comparison with a supportive unspecific intervention (SUI) of the same quantity in patients with LLD |
| **Time plan** | First patient first visit (FPFV): Q2/2018<br><br>Recruitment period: Q2/2018 – Q2/2020<br><br>Last patient last visit (LPLV): Q2/2020<br><br>Trial report: Q1/2021 |
| **Trial sites** | Number of trial sites: up to 7<br>Number of countries: 1 (Germany) |
| **Trial population** | To be assessed for eligibility: n = 300<br>To be allocated to trial: n = 248<br>To be analysed: n = 248 ITT, n =198 PP |
| **Treatment(s) / Intervention(s)** | Experimental intervention: A manual-based, individual,15-session, twice weekly, outpatient CBT specifically designed for patients with late life depression.<br><br>Control intervention: A manual-based 15-session, twice weekly, outpatient standard treatment containing supportive and educational elements (supportive unspecific intervention, SUI).<br><br>Duration of intervention per patient: 8 weeks.<br><br>Follow-up per patient: 6 months after randomization. |
| **Inclusion criteria** | Participating patients have to fulfil the following inclusion criteria:<br>• out-patient status<br>• male/female of age ≥ 60 years<br>• depressive episode (moderate to severe according to ICD-10)<br>• GDS > 10, QIDS-C > 10<br>• MMST > 25 points<br>• no or stable (≥ 6 weeks) antidepressive pharmacological treatment at baseline<br>• no or stable antidepressive pharmacological treatment during 8-week intervention<br>• sufficient German language skills |

| | |
|---|---|
| **Exclusion criteria** | Any of the following will exclude a patient from the trial:<br>• Bipolar depression<br>• Schizophrenia<br>• Other psychotic disorder<br>• Substance abuse or dependency<br>• Dementia<br>• Acute suicidality<br>• Anxiety disorder as stand-alone diagnosis (e.g. generalized anxiety disorder, panic disorder, social phobia)<br>• Obsessive-compulsive disorder (OCD) as stand-alone diagnosis<br>• Participation in any other clinical trial parallel to this trial<br>• Additional psychological/psychotherapeutic treatment throughout the treatment period<br>• Regular use with scheduled dosing of Benzodiazepines (not PRN)<br>• Severe medical condition, which clearly impacts on depression or on the ability to participate in the trial<br>• Brain disease with severe functional impairement that impacts the ability to participate in the trial (e.g. aphasia, Parkinson's disease) |
| **Outcome(s)** | The primary efficacy endpoint is the change in Geriatric Depression Scale (GDS) from baseline (T0) to end-of-treatment (T2).<br><br>Key secondary endpoint(s) is the change in Geriatric Depression Scale (GDS) from baseline to end of follow-up (T3) as well as changes from baseline to end-of-treatment (T2) and to end of follow-up (T3; 6 months after randomization) in:<br>• Quick Inventory of Depressive Symptomatology (QIDS-C)<br>• Geriatric Anxiety Inventory (GAI)<br>• Patient-Reported Outcome in Major Depressive Disorder (PRO-MDD)<br>• WHO Quality of Life (WHOQOL-OLD, WHOQOL-BREF)<br>• Health Status (SF 36)<br>• Insomnia Severity Index (ISI)<br>• Epworth Sleepiness Scale (ESS)<br>• REM Sleep Behavior Disorder Screening Questionnaire (RBDSQ)<br>• Consortium to Establish a Registry for Alzheimer's Disease neuropsychological battery (CERADplus)<br>• Subtest of the Neuropsychological Assessment Battery (NAB)<br><br>The influence of traumatic experiences, and personality traits on the change in GDS will be assessed at baseline by:<br>• Childhood Trauma Questionnaire (CTQ-SF)<br>• Big Five Inventory-10 (BFI-10)<br><br>The longitudinal evaluation of depressive symptoms will be conducted by the Longitudinal Interval Follow-up Evaluation (LIFE) at follow-up (T3).<br><br>Assessment of safety:<br>• AE and SAE recordings<br>• assessment of suicidality |
| **Schedule** | Screening and baseline visit (T0): week 1 (visit 1)<br>Treatment period: 8 weeks (15 sessions)<br>First Outcome assessment (T1): week 5 (visit 9, after 7 therapy sessions)<br>Second Outcome assessment (T2): week 10 (visit 18, after completion of 15 therapy sessions)<br>Follow-up period: 6 months (after randomization)<br>Final examination: 6 months after randomization (visit 19) |

| | |
|---|---|
| **Course of the trial** | Flowchart in section 12.1. |
| **Statistician** | Dr. rer. medic. Hildegard Christ<br>Institute of Medical Statistics and Computational Biology (IMSB)<br>University of Cologne<br>Kerpener Str. 62<br>50931 Cologne<br>Germany<br>Phone: +49 221 478 6502,<br>E-mail: hildegard.christ@uk-koeln.de |
| **Statistical Analysis** | Sample size: 300 (to be screened)<br>Trial population:<br>*Full-analysis-set population (FAS, Intention-to-treat population)*: 248<br><br>*Per-protocol-set population (PPS):*<br>198<br>(defined as a subset of the FAS population patients without major protocol violations.)<br><br>Efficacy / test accuracy: The evaluation is based on change on the GDS with a range of 0-30 points.<br><br>Description of the primary efficacy analysis and population: The primary (full) analysis set (FAS) is derived from the intention-to-treat principle (all subjects randomized with a valid baseline assessment and at least one valid follow-up outcome assessment). The change in GDS from baseline to end-of-treatment is evaluated by means of a mixed model for repeated measures (MMRM) with fixed effects group, time, group*time, therapist and baseline value; corresponding treatment contrasts are based on marginal means. To assess the impact of up to 20% attrition multiple imputation approaches are taken, accounting for proxy measures and assuming specific missingness-not-at-random patterns. The details are documented in a statistical analysis plan. Analysis of subjects essentially observed and treated per protocol (PP) is supportive. Subgroup analysis is done i.a. by gender and disease severity (moderate, severe).<br><br>Safety: Adverse events are listed and summarized by group and category, seriousness, severity, relatedness, respectively.<br><br>Secondary endpoints: Quantitative outcomes are summarized by mean, standard deviation and percentiles (i.e. 0, 25, 50, 75, 100), qualitative outcomes by count (percentage). Differences between treatment groups and changes over time are essentially evaluated along the same line as for the primary outcome measure, i.e. using mixed modeling (or GEEs). |
| **Financing** | Bundesministerium für Bildung und Forschung (BMBF)<br>DLR Projektträger für das BMBF<br>Gesundheitsforschung<br>Heinrich-Konen-Straße 1<br>53227 Bonn<br>(BMBF reference: 01KG1716) |

## 1.2 FINANCING OF TRIAL

The trial CBTlate is an investigator-initiated trial led by Prof. Dr. med. Frank Jessen as the Sponsor's representative. Financing for this trial has been secured by a grant from the "Bundesministerium für Bildung und Forschung" (BMBF) with the reference 01KG1716.

## 1.3 Table of contents (removed from here)

### 1.3.1 List of in-text tables

**Table 1**: Time schedule of the study

**Table 2:** Trial Schedule

**Table 3:** Intensity of adverse events

**Table 4:** Suicidality risk level

### 1.3.2 List of in-text figures

**Figure 1**: Flow chart of trial procedures

## 1.4 LIST OF ABBREVIATIONS

| **Abbreviation** | **Term** |
|---|---|
| AE | adverse event |
| BMBF | Bundesministerium für Bildung und Forschung |
| CA | competent authority |
| CBT | cognitive behavioral therapy |
| CRF | case report form |
| CRO | contract research organisations |
| CTCC | Clinical Trials Centre Cologne |
| DCF | data clarification form |
| DMSB | Data Monitoring and Safety Board |
| EC | ethics committee |
| ECT | Electroconvulsive Therapy |
| FAS | Full-analysis-set |
| GCP | Good Clinical Practice |
| IMSB | Institute of Medical Statistics and Computational Biology |
| ITT | Intention-to-treat |
| LLD | Late-life depression |
| LPI | Lead Principle Investigator |
| PP | Per Protocol |
| PPS | Per-protocol-set |
| SAE | serious adverse event |
| SAR | serious adverse reaction |
| SUI | supportive unspecific intervention |
| tDCS | Transcranial Direct-Current Stimulation |
| TMS | Transcranial Magnetic Stimulation |
| VNS | Vagus Nerve Stimulation |

## 1.5 ETHICS

### 1.5.1 Independent Ethics Committee

Independent Ethics Committees (EC) will safeguard the rights, safety, and well-being of all trial subjects.

In each trial site, the clinical trial will not be started before approval of the respective competent local ethics committee concerning the suitability of the trial site and the qualifications of the investigators.

### 1.5.2 Ethical conduct of the trial

This trial is to be conducted according to the ethical principles that have their origin in the Declaration of Helsinki and that are consistent with Good Clinical Practice (GCP) and applicable regulatory requirement(s). GCP is a standard for the design, conduct, performance, monitoring, auditing, recording, analyses and reporting of clinical trials that provides assurance that the data and reported results are credible and accurate, and that the rights, integrity and confidentiality of the trial subjects are protected.

All subjects will be informed that participation is voluntary and that they can cease participation at any time without necessarily giving a reason and without any penalty or loss of benefits to which they are entitled.

### 1.5.3 Legislation and guidelines used for preparation

The present clinical trial will be conducted in accordance with the published principles of the guidelines for Good Clinical Practice (ICH-GCP) and applicable legislation. These principles cover, amongst other aspects, ethics committee procedures, the obtaining of informed consent from trial subjects, adherence to the trial protocol, administrative documentation, data collection, trial subjects' medical records (source documents), documentation and reporting of adverse events (AEs), preparation for audits, and the archiving of trial documentation. All investigators and other staff directly concerned with the study will be informed that domestic supervisory bodies and authorised representatives of the sponsor have the right to review trial documentation and the trial subjects' medical records at any time.

### 1.5.4 Notification of the authorities, approval and registration

As the regulations of federal drug law (Arzneimittelgesetz, AMG) or Medical Products Act (Medizinproduktegesetz, MPG) do not apply to this trial, notification is not applicable.

Before the trial is started, it will be registered in the German Clinical Trials Register (Deutschen Register Klinischer Studien, DRKS, https://www.drks.de), approved by the World Health Organisation (WHO, http://www.who.int/ictrp/en/). Furthermore, the trial will be registered at ClinicalTrials.gov. The trial protocol will be submitted for publication.

### 1.5.5 Subject information and consent

With the help of the information sheet the subject will be informed about the treatment. At the same time the subject will be informed of the reason, design and implication of the trial. The subject must give his consent to participate prior to any trial specific investigations which includes the screening examination. This consent must be given in writing. The Investigator who conducted the informed consent discussion must also sign. With his consent the subject will confirm that his participation is voluntary and that he will follow the instructions of the Investigator and answer the questions that are asked of him.

Prior to participation in the trial, the subject will receive a copy of the signed and dated written informed consent form and any other pertinent written information. Neither the Investigator, nor the trial staff, should coerce or unduly influence a subject to participate or to continue to participate in the trial. Ample time *(the length of which depends on local regulations)* must be allowed for the subject to make his or her decision, and to make further enquiries about the trial. The signed consent form will be kept by the Investigator.

### 1.5.6 Insurance

Insurance for the subjects included in this trial will be arranged by the University of Cologne, Department of Psychiatry according to local requirements. Also, a travel accident insurance is provided for the trial subjects. The headquarters, policy number and telephone/fax number will be included in the patient informed consent form. A copy of each insurance certification will be held in the trial master file at CTCC and by the Clinical Project Manager at the University of Cologne, Department of Psychiatry.

### 1.5.7 Subject data and data protection

The provisions of data legislation will be observed. It is assured by the Lead Principle Investigator that all investigational materials and data will be pseudonymized in accordance with data protection legislation before scientific processing.

Trial subjects will be informed that their pseudonymized data will be passed on in accordance with provisions for documentation and notification to the recipients decribed there.

It is the Clinical Research Associate's responsibility to verify that trial subjects have consented in writing according to the Monitoring Plan. Subjects who do not agree that the information may be passed on in this way will not be enrolled into the trial.

### 1.5.8 Point of contact

As required by national regulations subjects will be provided with information on a contact point where additional information can be obtained about the trial and the right of the subject and whom to contact in the event of trial-related injury. This source of information will also be specified in the information sheet and Informed consent form.

## 1.6 TRIAL ADMINISTRATIVE STRUCTURE

**Sponsor:** University of Cologne, Albertus-Magnus-Platz, 50923 Cologne , Germany

Represented by: Prof. Dr. med. Frank Jessen, Department of Psychiatry and Psychotherapy, University of Cologne Medical Faculty, Kerpener Str. 62, 50937 Cologne, Germany, Tel.: +49-221-478-4010, Fax: +49-221-478-5593, E-mail: frank.jessen@uk-koeln.de

**Lead Principal Investigator:** Prof. Dr. med. Frank Jessen, Department of Psychiatry and Psychotherapy, University of Cologne, Medical Faculty, Kerpener Str. 62, 50937 Cologne, Germany, Tel.: +49-221-478- 4010, Fax: +49-221-478-5593, E-mail: frank.jessen@uk-koeln.de

**Co-Lead Principal Investigator:** Prof. Dr. phil. Martin Hautzinger, Department of Psychology, Clinical Psychology and Psychotherapy, Eberhard-Karls-University, Schleichstrasse 4, 72076 Tuebingen, Germany, Tel. +49-7071-2977301, Fax: +49-7071-295219, Email: hautzinger@uni-tuebingen.de

**Statistician:** Dr.rer.medic. Hildegard Christ, Institute of Medical Statistics and Computational Biology (IMSB), University of Cologne, Kerpener Str. 62, 50931 Cologne, Germany, Phone: +49 221 478 6502, E-mail: hildegard.christ@uk-koeln.de

**Data Monitoring and Safety Board (DMSB):** Prof. Dr. med. Stefan Teipel , Department of Psychosomatic Medicine, University of Rostock, Gehlsheimer Strasse 20, 18147 Rostock ,Germany

Prof. Dr. Gabriele Wilz, Department of Psychology, University of Jena, Humboldtstrasse 11, 07743 Jena, Germany

Prof. Dr. Wolfgang Köpcke, Institute of Clinical Research and Biometry, Department of Psychology, University of Muenster, Schmeddingstr. 56, 48149 Muenster, Germany

**Central organisation units:**

The Lead Principal Investigator as the Sponsor‘s representative delegates all administrative and non-clinical project management tasks for executing the trial project management, monitoring, data management, safety management to the Clinical Trials Center Cologne.

Clinical Trials Centre Cologne, Gleueler Str. 269, 50935 Cologne, Phone: +49 221 478 88121, Fax: +49 221 478 88209

**Investigators and trial sites:**

The trial will be carried out as a national multi-center trial at several trial sites in Germany. All participating investigators signed the declarations of commitment of participating centers. Sponsor will conclude separate Clinical Trial Agreements with all participating centers. If necessary, further qualified trial sites may be recruited to the trial.

A listing of trial sites, principal investigators, sub-investigators, and further trial staff, will be kept and continuously updated in a separate list. The final version of this list will be attached to the final report of the clinical trial. Only investigators and participating trial sites are selected for the trial that meet the regulatory requirements with qualification and experience to perform a clinical investigation including trials of pharmaceutical preparations. The sponsor will appoint the principal investigator at each study site, who in turn, will select qualified and experienced staff for trial conduct.

## 1.7 SCIENTIFIC BACKGROUND AND RATIONALE

Depressive disorders are frequent health problems that are among the leading causes of disability worldwide [44]. With the growing population aged 60 years and older and the longer life expectancy of individuals, the demand on mental health care services by the geriatric population is expected to increase. Depression is one of the most prevalant mental disorders in old age [41]. Late-life depression (LLD) is generally characterized as a depressive episode occuring past the age of 60-65. Metaanalysis of epidemiological data suggest a prevalence rate of 7.2% (95 % CI 4.4-10.6%) for major depression and 17.1% (95% CI 9.7-26.1%) for depressive disorders in the elderly population [28]. The prevalence is about twice as high in women compared with men [7; 28]. According to a German longitudinal study, the incidence of LLD is 3.7 in men and 4.6 in women per 100 person-years [42]. A review on international studies reported incidence rates of LLD of 0.2-14.1/100 person-years [6].
Late-life depression is associated with various adverse outcomes such as reduced quality of life, negative impact on physical comorbidities, functional impairments, increased suicide and non-suicide mortality [10]. Despite the lower rates of depression among older adults, suicide rates are higher in this age group than in any other group. While suicide occurs in 10.2/100.000 individuals in Germany below the age of 65, it increases to 25.7/100.000 after the age of 65 [39]. Moreover, LLD serves as a risk factor for developing all-cause dementia, including Alzheimer´s disease [1; 31; 32; 38]. Furthermore, Depression characteristically complicates the course and outcome of other medical illnesses among older adults such as cardiovascular disease, diabetes mellitus, cancer and infectious disease. LLD increases the incidence of other medical conditions, accelerating the respective disease process and worsening outcome [12]. Due to the negative impact of LLD on the course and outcome of medical diseases, LLD leads to a frequent use of health care services. It increases the socioeconomic burden of all of these medical conditions and remains a serious public health concern. Prospective analysis revealed that the health care costs of elderly individuals with depression are one third higher than those of non-depressed subjects [29; 30].
Unfortunately, LLD is often misinterpreted as a physiological aging process and thus remains under-reported, under-recognized, misdiagnosed, and under-treated for years [23; 34]. Compared with depression in early adulthood, treatment options of LLD are limited. Antidepressants are less effective in LLD than in depression in young and in middle age adults. The number-needed-to-treat (NNT) for remission of depression by antidepressant

medication is around 7 in patients younger than 65 years of age [3] and increases to 14.4 in subjects older than 65 [25]. Also, side effects, intolerability and contraindications of antidepressants increase with age, which limits their application.

Psychotherapy is associated with less risk and potentially greater benefit in LLD than medication. A highly established and effective type of psychotherapy for depression is cognitive behavioural therapy (CBT), of which efficacy has been demonstrated in young and middle age adults [2]. To be effective in LLD, however, it needs to be adapted to the specific characteristics and needs of the older age patient group, which are distinct from young and middle-aged adults (e.g. loss of close others, loneliness, retirement, physical impairment, financial restraints, closeness of death). The evidence of efficacy of LLD-adapted CBT is very limited. The vast majority of studies on CBT in LLD is of insufficient power or has other significant methodological shortcomings so that they either remain non-conclusive or are not generalizable. In a meta-analysis Wilson et al. [43] included 3 studies, which compared CBT, as defined by the authors, against an active control group. Of these studies, only one applied a technique, which corresponds to one component of the current conceptualization of CBT (problem solving) but only included the limited number of 25 patients per arm [43]. Gould et al. included 21 studies in their meta-analysis [13]. Of these, only 5 included more than 50 patients in total and only one of these 5 tested CBT against an unspecific control intervention [36], while the rest tested against a waiting list control group. Comparison against a control intervention, however, is crucial to distinguish CBT-related effects from unspecific effects of intensified patient management and placebo response. Thus, waiting list control conditions are not sufficient to establish efficacy of a specific psychotherapeutic intervention. The study with an active control group was a single-centre study with recruitment in the primary care setting and inclusion of self-referral patients, which also introduces bias with regard to the real world clinical population of the psychiatric care setting [36]. In a recent systematic review on psychological intervention in LLD, 5 studies on CBT were included, which reached the pre-specified level of quality (risk of bias) [22]. Of these 5 studies, 3 included more than 50 patients in total. In 2 of these 3 studies, patients with a major depressive disorder (MDD) were excluded so that the studies focused on individuals with mild depressive symptoms only. In a pilot study the short- and long-term outcome of a manualized LLD-specific CBT-intervention in comparison to a manualized supportive, but unspecific intervention (SUI), delivered either in an individual or a group setting was tested [19]. Older adults with depression had been randomized to one of four interventions (CBT-individual, CBT-group, SUI-individual, SUI-group) with a maximum sample size per arm of n = 31. Outcomes were assessed by self- and clinician ratings before the 15-session interventions, at the end and after one year of follow-up. CBT reduced depressive symptoms significantly more than SUI. This effect was greatest in the CBT-arm with individual treatment (not group treatment) and the effect extended over one year.

Based on these results, there is promising evidence for CBT in LLD, but the quality of evidence is low due to the limited number of studies. Yet, an LLD-specific CBT with recruitment in the specialized clinical (psychiatric/psychotherapeutic) setting in patients with moderate to severe LLD has never been compared to an unspecific supportive psychological intervention in a multicentre study.

In summary, the available data support the need for a large, multicenter trial including an individually delivered LLD-specific CBT to aim at establishing a confirmatory evidence for the efficacy of this individually delivered specific cognitive behavioral intervention for late-life depression.

This study addresses the unmet medical problem of insufficient treatment of late life depression (LLD). It is the largest study and first confirmatory multicentre trial to test the efficacy of an LLD-adapted cognitive behavioural therapy (CBT) program. The target population will be patients with moderate to severe disease, recruited from the psychiatric setting. This will ensure targeting the most relevant patient group and is distinct from earlier studies, which included self-referred patients or only patients at the mild disease stage. If effective, this treatment could relieve the burden of depression in terms of reducing depression associated morbidity and mortality, reducing the negative impact of depression on several other medical conditions and improving quality of life for this patient group. The socioeconomic benefit would be a reduction of LLD-related long-term treatment costs and a reduction of costs associated with several medical comorbidities, which are negatively affected by LLD with regard to course and outcome. These potential effects are of particular importance in the rapidly growing patient group above the age of 60 years.

### 1.7.1 Benefit/risk analysis

The study is not expected to harm any patient or include significant risk for the patient. The individual taking part in this trial will either receive the full LLD-CBT program or the SUI control intervention, which will both provide

benefit and represent substantially more treatment than regular health care. The study is a highly beneficial offer for LLD patients because it provides treatment in both arms, which is usually not provided to these patients in standard care. The risk for the participating individual during the diagnostic procedures or outcome assessments is minimal. The intervention is of limited length and is expected to improve outcome. None of the interventions will confront the patients with negative contents, such as reactivation of trauma as an example. In contrary, both interventions are supportive and aim at stabilizing the mental status of the patients. The interventions will be provided by experienced psychotherapists, which will also limit the risk of harm. The Investigators will ensure that the trial is conducted in complete conformance with the principles of Good Clinical Practice (GCP) and the Declaration of Helsinki. Still, adverse events and serious adverse events will be recorded at every visit and will be reported to a data safety and monitoring board (DSMB) on a continuous basis. Although side effects of evidence-based psychotherapies such as CBT are very rare, possible undesired effects may include a transient worsening of symptoms or risk of suicidality. For any signs or symptoms or adverse events, a causal or symptomatic treatment according to standard medical practice is performed, if deemed necessary by the Investigator.

## 1.8 STUDY OBJECTIVES AND ENDPOINT CRITERIA

### 1.8.1 Objectives

#### 1.8.1.1 Primary Study Objectives

The primary objective of this study is to test the hypothesis that a 15-session individually-delivered cognitive behavioural therapy (CBT) specific for late life depression (LLD) is of superior efficacy in reducing symptoms of depression in comparison with a supportive unspecific intervention (SUI) of the same quantity in subjects with LLD.

#### 1.8.1.2 Secondary Study Objectives

The secondary objective of this study is to test the efficacy of LLD-CBT in comparison with SUI on patient reported outcome in major depressive disorder, anxiety, sleep, cognition, quality of life and overall health status. Furthermore, we will be investigating the influence of childhood traumatic experiences and personality traits on the change of depressive symptoms in LLD-CBT in comparison to SUI.

### 1.8.2 Endpoint criteria

#### 1.8.2.1 Primary endpoint criteria

The primary efficacy endpoint is the change in Geriatric Depression Scale (GDS, range 0-30) from baseline to end-of-treatment [45], which is a widely established instrument to measure symptoms of depression in older subjects and to measure effects of treatment in clinical trials. It has also been employed as the primary endpoint in the pilot study of this trial [19]. It is a self-rating scale including 30 Items in a yes/no format, which measures the subject related effect of the intervention. The GDS score can range from 0 to 30.

#### 1.8.2.2 Secondary endpoint criteria

The key secondary endpoints are the change in Geriatric Depression Scale (GDS) from baseline to end of follow-up (T3) as well as changes from baseline to end-of-treatment (T2) and to end of follow-up (6 months after randomization) in:

- Quick Inventory of Depressive Symptomatology (QIDS-C)
- Geriatric Anxiety Inventory (GAI)
- Patient-Reported Outcome in Major Depressive Disorder (PRO-MDD)
- WHO Quality of Life (WHOQOL-OLD, WHOQOL-BREF)

- Health Status (SF 36)
- Insomnia Severity Index (ISI)
- Epworth Sleepiness Scale (ESS)
- REM Sleep Behavior Disorder Screening Questionnaire (RBDSQ)
- Consortium to Establish a Registry for Alzheimer's Disease neuropsychological battery (CERADplus)
- Subtest of the Screening Module (Executive Funtions: Mazes) of the Neuropsychological Assessment Battery (NAB)

Furthermore, we will be investigating the influence of childhood traumatic experiences and personality traits on the change in depressive symptoms in LLD-CBT in comparison to SUI by the following instruments at baseline:

- Childhood Trauma Questionnaire (CTQ-SF)
- Big Five Inventory-10 (BFI-10)

The longitudinal evaluation of depressive symptoms will be conducted at follow-up by assessing the outcomes in the Longitudinal Interval Follow-up Evaluation (LIFE).

All of the listed measures provide information of clinical relevance for the target population. All have been validated. As an exploratory outcome, qualitative interviews will be performed at the end of the treatment period to assess individuals' needs and goals met and not met by the intervention. All primary and secondary outcome measures are scored according to the respective manuals (global score, sums of items, score in subscales).

## 1.9 TRIAL DESIGN AND METHODOLOGY

### 1.9.1 Trial design

This is a randomized, multi-center, single-blind (observer-blinded), active-controlled, parallel group trial.

The planned number of subjects is 300 subjects assessed for eligibility and 248 allocated to the trial. The planned number of subjects per treatment (LLD-CBT, LLD-SUI) is 124.
Participating sites have been selected on the basis of being highly experienced in old age psychiatry, in the delivery of psychotherapy and in the running of clinical trials. All participating sites have outpatient clinics with several hundred patient contacts per year, including high numbers of patients with LLD. In addition, participating sites collaborate with networks of psychiatrist and psychotherapist, which may be involved in patient identification, if needed.

### 1.9.2 Discussion of trial design

*Selection bias* is minimized by randomly assigning subjects to treatment groups (ratio 1:1) according to permuted blocks of varying length. Randomization will be stratified by site in order to prevent unbalanced allocation of the intervention of interest and the control intervention to sites. It will be implemented as a central 24-7 Internet service operated by local, authorized study staff. Treatment assignments will be displayed on screen and delivered by e-mail. The randomization service will be maintained by the IMSB, University of Cologne.
*Performance bias* is minimized by carefully following the study protocol and continuous monitoring of adherence. Both the experimental and the control intervention are manual-based and are provided by the same therapist, trained in both programs. The sessions are filmed and the Co-Lead Principal Investigator (M. Hautzinger) assures adherence to the manuals in both treatment conditions (see section 12.3.3). The outcome assessments performed by the blinded rater will be filmed as well to assure adherence to protocol.
*Attrition bias* is minimized by dedicated follow-up, nevertheless, up to 20% attrition are expected due to the specific late life care setting. The attrition rate in the pilot study at the 5-months follow-up was 11% (data provided by M. Hautzinger) [19]. Proxy measures (e.g. from care givers) are taken into account to alleviate the corresponding risk of bias.

*Detection bias* is minimized by masking the researchers who collect data independent of the clinical team, i.e. study participants will be asked not to tell the researcher whether they are in the intervention or in the standard care group, which is the standard approach in clinical trials on psychotherapy. Moreover, the analyzing statistician(s) will be kept masked until database lock.
The following measures of bias are used:

1. Expected outcome (prior to the study, all therapists are asked to predict the degree to which they think either treatment would do harm or good to each subject on a 7-point Likert scale ranging from −3 to +3)
2. Subjects' preferences for treatment, recorded on a 4-point Likert scale (scores, 0-3)
3. Assessment of blindness (the researcher undertaking assessment is asked to guess the trial group [yes, no, don't know]) [36].

### 1.9.3 Blinding

This is a single-blind (observer-blinded) trial. The primary and secondary outcomes will be obtained at baseline, in week 5 after 7 treatment sessions, in week 10 at the end of treatment and at follow-up (6 months after randomization) by the rater, who will be blinded to the treatment arm allocation. After the baseline visit, the participants will be randomized to either of the two treatment arms (1:1 randomization, see section 15.2.).

### 1.9.4 Time schedule

**Table 1:** Time schedule (estimated)

| | |
|---|---|
| Duration of trial: | 36 months |
| Recruitment period: | Q2/2018 – Q2/2020 |
| First patient first visit (FPFV): | Q2/2018 |
| Last patient last visit (LPLV): | Q2/2020 |
| Data base clearing/analysis: | Q2/2020 – Q3/2020 |
| Trial report: | Q1/2021 |
| Period of time for patient in trial: | 6 months |
| Screening period: | 1 week |
| Treatment period: | 8 weeks |
| Follow-up period: | 6 months (after randomization) |

### 1.9.5 End of trial

The end of trial is defined as the last visit of the last subject (subject number 248).

## 1.10 TRIAL POPULATION

### 1.10.1 Definitions

Subject definitions and subject completion:

- **Enrolled** subjects are those on whom trial specific investigations are performed after informed consent is given.
- **Randomized subjects / subjects assigned to treatment** are those allocated to one of the therapeutic interventions.
- **Treated** subjects are those who have completed at least 9 sessions in one of the therapeutic intervention as scheduled in the trial protocol.
- A **"completed"** subject is one who has completed all sessions of the therapeutic intervention and the follow-up session as scheduled in the trial protocol.

- A **“drop-out”** is an enrolled subject who stops prematurely before session 9 of the intervention. This can be due to non-observance of the protocol or to reasons unrelated to the trial, e.g., “moved away” or who are otherwise lost before final examination as scheduled. A subject who simply wishes to terminate the trial should also be considered as a drop-out.
- A **“withdrawal”** is an enrolled subject who stops prematurely or is withdrawn by the Investigator for reasons related to the trial, e.g., an adverse event.

### 1.10.2 Selection of trial population

#### 1.10.2.1 Recruitment procedure

Subjects will be recruited consecutively if they suffer from late-life depression, fulfill the inclusion criteria, do not meet any exclusion criteria and have signed the informed consent.

Participating sites have been selected on the basis of being highly experienced in old age psychiatry, in the delivery of psychotherapy and in the running of clinical trials. All participating sites have outpatient clinics with several hundred patient contacts per year, including high numbers of patients with LLD. In addition, all sites collaborate with networks of psychiatrist and psychotherapist, which may be involved in patient identification. Further, advertisements will be placed at collaborating resident general and psychiatric practices.

#### 1.10.2.2 Screening log

Each participating center will keep a log of all screened subjects with LLD.

#### 1.10.2.3 Inclusion criteria

Participating subjects have to fulfil the following criteria:

| **Inclusion Criteria** |
|---|
| • out-patient status |
| • male/female, age ≥ 60 years |
| • depressive episode (moderate to severe according to ICD-10) |
| • Geriatric Depression Scale (GDS) > 10 |
| • Quick Inventory of Depressive Symptomatology (QIDS-C > 10) |
| • MMST > 25 points |
| • no or stable (≥ 6 weeks) antidepressive pharmacological treatment at baseline |
| • no or stable antidepressive pharmacological treatment during 8-week intervention |
| • sufficient German language skills |
| • written informed consent signed |

#### 1.10.2.4 Exclusion criteria

Any of the following will exclude a subject from the trial:

| **Exclusion criteria** |
|---|
| • Bipolar depression |
| • Schizophrenia |
| • Other psychotic disorders |
| • Substance abuse or dependency |
| • Dementia |
| • Acute suicidality |
| • Anxiety disorder as stand-alone diagnosis (e.g. generalized anxiety disorder, panic disorder, social phobia) |

| Exclusion criteria |
| --- |
| • Obsessive-compulsive disorder as stand-alone diagnosis |
| • Participation in any another clinical trial parallel to this trial |
| • Additional psychological/psychotherapeutic treatment throughout the treatment period |
| • Regular use with scheduled dosing of Benzodiazepines (not PRN) |
| • Severe or instable medical condition, which clearly impacts on depression or on the ability to participate in the trial |
| • Brain disease with severe functional impairement that impacts the ability to participate in the trial (e.g. aphasia, Parkinson's disease) |

Any of the following will exclude a subject from the Magnetic Resonance Imaging (MRI) acquisition, if applicable:

| Exclusion criteria |
| --- |
| • History of cardiac pacemaker, defibrillator or neurostimulator implantation |
| • Insulin or infusion pump |
| • Cochlear, otologic or ear implant |
| • Any implant held in place by a magnet |
| • Tissue expanders (plastic surgery) |
| • Implanted catheter, clamp, clips, valves, or other metal |
| • Tattoos or permanent makeup |
| • History of metalworking |
| • Injury with shrapnel or metal slivers |
| • Injury to the eye involving metallic object or fragment |
| • Recent major surgery |
| • History of major head trauma with loss of consciousness |
| • Neurological disease (e.g. epilepsy, stroke, Parkinson's disease, multiple sclerosis) |
| • Untreated or unstable cardiovascular, pulmonary, liver or kidney disease (e.g. unstable hypertension or cardiac arrythmias) |
| • Claustrophobia |
| • Weight > 160 kg |

### 1.10.3 Removal of subjects from the trial

A subject may terminate participation in the trial at any time without providing a reason and without any personal disadvantage. Additionally, the Investigator can stop the participation of a subject after consideration of the benefit/risk ratio. Other possible reasons for the Investigator to discontinue a subject's trial treatment or participation include:

- Any relevant deterioration in the health of the subject possibly impacting the participation in the trial, including adverse events, severe medical condition, major surgery, laboratory parameters, vital signs or other safety parameters at the discretion of the Investigator.
- Regular use with scheduled dosing of Benzodiazepines (not PRN) or change of antidepressive pharmacological treatment during 8-week intervention.
- Parallel psychiatric or psychotherapeutic interventions (including but not limited to ECT, TMS, tDCS, VNS) during the treatment period.
- Technical grounds (e.g. subject moves away).

The Investigator must prepare a detailed written explanation for the LPI and the EC/IRB where applicable. The LPI and CTCC should be informed within three business days about the withdrawal of a subject from the trial.

In the single centre pilot study (data provided by M. Hautzinger) [19], the attrition rate was 11% at the follow-up visit at six months after the end of the treatment. In this multicentre trial an attrition rate of 20% has been estimated, which seems realistic since the population is highly similar to that of the pilot study.

#### 1.10.3.1 Notification of discontinuations

The LPI and CTCC must be informed within three business days about any removal of a subject from the trial including the reason. The Investigator must prepare a detailed written explanation for the LPI.

### 1.10.4 Trial completion

#### 1.10.4.1 Subject definition and subject completion

See Section 11.1.

#### 1.10.4.2 Procedures for handling of replacements, withdrawals or drop-outs

For all subjects who terminate the trial prematurely the Investigator must compile a precise written documentation including the reason for premature termination. Subjects who withdraw or stop treatment prematurely (drop-out, withdrawal) will not be replaced. The Investigator should make the attempt to monitor patients after drop-out and, if feasible, to conduct the follow-up outcome assessments.

#### 1.10.4.3 Premature termination of the trial

The trial will be stopped for the individual study participant, if the individual:

- Withdraws the informed consent.
- Develops a medical condition, which prevents participation in the trial.
- Experiences worsening of depression, which requires inpatient treatment or change of antidepressive medication.
- Develops acute suicidality.

The trial may be prematurely terminated by the respective competent local EC or the Lead Principal Investigator, if:

- The perception of the benefit/risk ratio becomes unfavourable for the continuation of the trial.
- The risk-benefit balance for the trial subject changes markedly.
- It is no longer ethical to continue treatment.
- It is no longer practicable to complete the trial.

However, our trial and study protocol are not to be expected to harm any subject but to improve outcomes. We do not expect that the trial has to be stopped in advance.

A decision to cease the trial is binding on all Investigators. Clinical Trials Centre Cologne, as well as the principal Investigators in each centre, must be informed immediately on the discontinuation of the trial. If the trial is prematurely terminated or suspended for any reason, the Investigator should promptly inform the subjects, should assure appropriate follow-up for the subjects, and inform the institution where the trial was being performed.

Participating centres, which fail to include the estimated number of subjects or to guarantee treatment in accordance to study protocol, will be closed.

## 1.11 TRIAL PROCEDURES

### 1.11.1 Flow chart

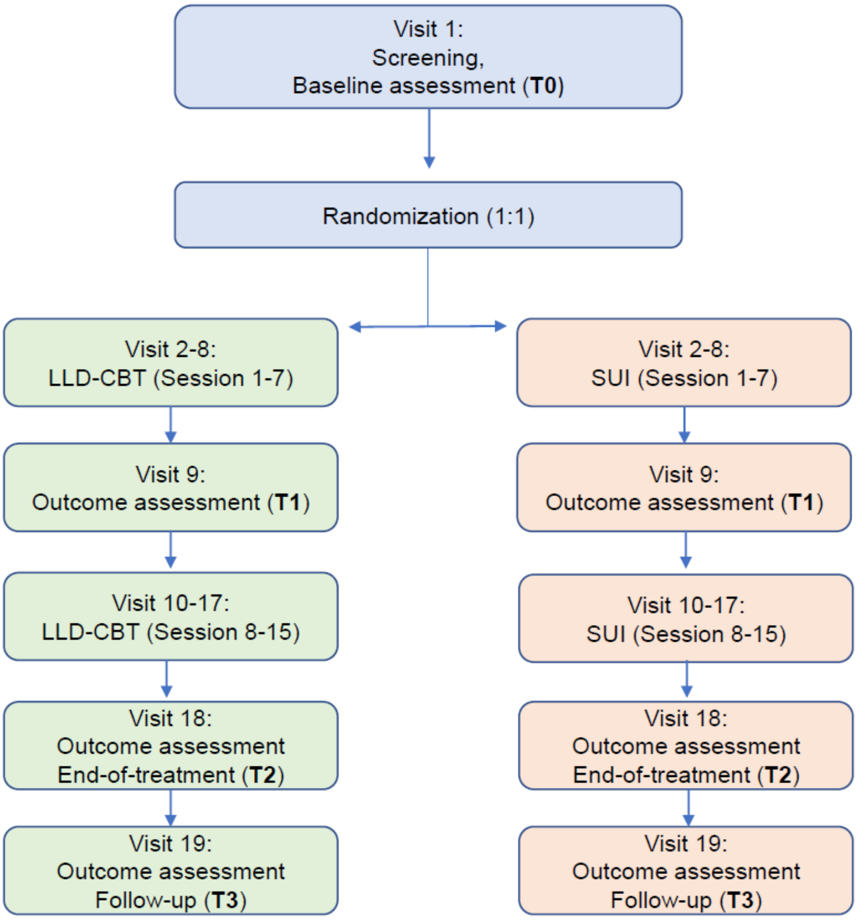


**Figure 1**: Flow chart of trial procedures

### 1.11.2 Trial schedule

**Table 2**: Trial Schedule detailing the trial procedures

| | **TRIAL SCHEDULE** | | | | | | | | | | | | | | | | | | | |
|---|---|---|---|---|---|---|---|---|---|---|---|---|---|---|---|---|---|---|---|---|
| **Procedure** | | | | | | | | | | | | | | | | | | | | |
| | **Screening** | **Baseline** | **Treatment** | | | | | | | | | | | | | | | | **End of treatment** | **Follow-up** |
| | **SCR** | **T0** | | | | | | | | **T1** | | | | | | | | | **T2** | **T3** |
| **Visit** | **1** | **1** | **2** | **3** | **4** | **5** | **6** | **7** | **8** | **9** | **10** | **11** | **12** | **13** | **14** | **15** | **16** | **17** | **18** | **19** |
| Week (W)<br>Month (M) | W1 | W1 | W1 | W2 | W2 | W3 | W3 | W4 | W4 | W5 | W5 | W5 | W6 | W6 | W7 | W7 | W8 | W8 | W10 | M6 |
| Written informed consent obtained | ● | | | | | | | | | | | | | | | | | | | |
| Evaluation of in-/ exclusion criteria | ● | | | | | | | | | | | | | | | | | | | |
| Demographic data | | ● | | | | | | | | | | | | | | | | | | |
| Concomitant diseases/ medication [a)] | ● | | | | | | | | | | | | | | | | | | | |
| Medical history | | ● | | | | | | | | | | | | | | | | | | |
| Structured clinical interview (MINI 7.0.2) | ● | | | | | | | | | | | | | | | | | | | |
| GDS | ● | ●* | | | | | | | | ● | | | | | | | | | ● | ● |
| QIDS-C | ● | ●* | | | | | | | | ● | | | | | | | | | ● | ● |
| MMST | ● | | | | | | | | | | | | | | | | | | | |
| GAI | | ● | | | | | | | | ● | | | | | | | | | ● | ● |
| PRO-MDD | | ● | | | | | | | | ● | | | | | | | | | ● | ● |
| WHO-QOL BREF & OLD | | ● | | | | | | | | ● | | | | | | | | | ● | ● |
| SF36 | | ● | | | | | | | | ● | | | | | | | | | ● | ● |
| CTQ-SF | | ● | | | | | | | | | | | | | | | | | | |
| BFI-10 | | ● | | | | | | | | | | | | | | | | | | |
| ISI/ESS/RBDSQ | | ● | | | | | | | | ● | | | | | | | | | ● | ● |
| CERADplus | | ● | | | | | | | | | | | | | | | | | | ● |
| NAB subtest maze | | ● | | | | | | | | | | | | | | | | | | ● |
| LIFE | | | | | | | | | | | | | | | | | | | | ● |
| Treatment (CBT or SUI) | | | ● | ● | ● | ● | ● | ● | ● | | ● | ● | ● | ● | ● | ● | ● | ● | | |
| Evaluation of SAEs, AEs [b)], exclusion criteria | | | ● | ● | ● | ● | ● | ● | ● | ● | ● | ● | ● | ● | ● | ● | ● | ● | ● | ● |
| Assessment of medical history | | | | | | | | | | | | | | | | | | | | ● |
| Voluntary procedures: | | | | | | | | | | | | | | | | | | | | |
| Blood sampling [c)] | | ● | | | | | | | | ● | | | | | | | | | ● | ● |

| | TRIAL SCHEDULE | | | | | | | | | | | | | | | | | | | |
|---|---|---|---|---|---|---|---|---|---|---|---|---|---|---|---|---|---|---|---|---|
| **Procedure** | | | | | | | | | | | | | | | | | | | | |
| | **Screening** | **Baseline** | **Treatment** | | | | | | | | | | | | | | | | **End of treatment** | **Follow-up** |
| | **SCR** | **T0** | | | | | | | | **T1** | | | | | | | | | **T2** | **T3** |
| **Visit** | **1** | **1** | **2** | **3** | **4** | **5** | **6** | **7** | **8** | **9** | **10** | **11** | **12** | **13** | **14** | **15** | **16** | **17** | **18** | **19** |
| MRI [d)] | | ● | | | | | | | | | | | | | | | | | ● | ● |

| | | |
|---|---|---|
| **a)** | **Concomitant medication** | Recording of any concomitant medication after signature of informed consent at baseline. |
| **b)** | **SAEs, AEs** | Recording of any spontaneously reported or observed SAEs and AEs after signature of informed consent until end of trial. |
| Voluntary procedures (after separate consent is given): | | |
| **c)** | **Blood sampling** | The study physician will take 4 tubes of venous blood (max. 50 ml in total per visit) from the subject at T0, T1, T2 and T3. The blood analyses include:<br>• Genetic and epigenetic analyses<br>• Measurement of Amyloid-β and Neurofilament light chain (NFL)<br>• miRNA analyses<br>• Metabolomics and Proteomics<br>• PBMC analyses<br>The total volume of the blood draws at each session (T0, T1, T2, T3) will not exceed 50 ml (see appendix document ‘Informationsbogen und Einwilligung CBTlate - Blutuntersuchung‘). |
| **d)** | **MRI** | A Magnetic Resonance Imaging (MRI) acquisition of the subjects’ brain will be performed at T0, T2 and T3 to obtain a high-resolution structural image (T1, T2 FLAIR) and a resting-state fMRI of the subjects’ brain before the psychotherapeutic intervention, at end-of-treatment and at follow-up. It will be complemented by Diffusion Tensor Imaging (DTI) of the subjects’ brain (see appendix documents ‘Informationsbogen zur MRT in der Studie CBTlate‘, ‘Einverständniserklärung MRT zur Studie CBTlate‘, ‘Selbstauskunft MRT zur Studie CBTlate‘). |
| ***** | **GDS/QIDS-C at T0** | Only needed if T0 is performed later than one week (7 days) after SCR. |

### 1.11.3 Course of the trial

The first visit of the study will be a screening/enrollment and baseline (T0) visit. The screening and baseline assessment can either be performed in a combined visit or in two separated visits (screening and baseline) within the same week for relief of the subjects.

Prior to screening the subjects will be informed about the study and the informed consent will be obtained. The inclusion and exclusion criteria as well as the primary outcome will be assessed. Only in those subjects who pass the screening procedure the secondary outcomes (baseline, T0) will be obtained.

The enrollment into the trial prior to screening will be performed by the study psychologist or physician. The study physician will obtain separate consent for participation in blood & MRI testing, if applicable. The outcomes will be obtained by the rater, who will be blinded to the later treatment arm allocation.

After the baseline assessment (T0), the subjects will be randomized to either of the two treatment arms (1:1 randomization). Within one week, the first of the successive bi-weekly 50-minute individual face-to-face treatment sessions will be performed by a study therapist.

After 7 therapy sessions, the primary and secondary outcomes will be obtained in week 5 (T1) by the blinded rater. This will be followed by the treatment sessions 8 to 15 which will be carried out by the therapist. End-of-treatment primary and secondary outcomes will be obtained in week 10 (T2) after completion of all 15 therapy sessions. The final follow-up assessment (T3) will be performed 6 months after randomization at the follow-up visit by the blinded rater.

#### 1.11.3.1 Information of subjects and informed consent

The subjects will be informed about the trial as well as the optional procedures, if applicable, at the first visit of the study. Before enrollment of a subject, the investigator will explain to each subject the nature of the trial, its purpose, the procedures, potential risks and benefits, confidentiality and privacy agreements as well as insurance cover. Each subject will be informed that participation is voluntary and withdraw is possible at any time. They will obtain information on the study in written form. Only subjects, who can provide full informed consent, will be included in the study. Full confidentiality with regard to study events will be provided. Written consent is obtained from the subjects.

#### 1.11.3.2 Enrollment and enrollment visit

The screening/enrollment visit includes the information of subjects and informed consent, the assessment of demographic data, concomitant medication and diseases. Furthermore, following measurements will be obtained in order to assess the inclusion and exclusion criteria:

- Structured clinical interview (M.I.N.I. for DSM-5, Mini International Neuropsychiatric Interview German Version 7.0.2)
- Geriatric Depression Scale (GDS)
- Quick Inventory of Depressive Symptomatology Clinician-Rated Version (QIDS-C)
- Mini-Mental-Status-Test (MMST)

Subjects who are eligible to participate in the study will undergo an assessment of the medical history, psychiatric and psychotherapeutic history, history of cognition (subjective cognitive decline) and the following procedures at baseline (T0):

a) *Questionnaires:*

- Geriatric Anxiety Inventory (GAI)
- Patient-Reported Outcome in Major Depressive Disorder (PRO-MDD)
- WHO Quality of Life (WHOQOL-OLD, WHOQOL-BREF)
- Health Status (SF 36)

- Insomnia Severity Index (ISI)
- Epworth Sleepiness Scale (ESS)
- REM Sleep Behavior Disorder Screening Questionnaire (RBDSQ)
- Childhood Trauma Questionnaire (CTQ-SF)
- Big Five Inventory-10 (BFI-10)
- Consortium to Establish a Registry for Alzheimer's Disease neuropsychological battery (CERADplus)
- Subtest of the Neuropsychological Assessment Battery (NAB) Screening Module (Executive Funtions: Maze)

The outcomes will be assessed by the rater, who will be blinded to the later treatment arm allocation.

Further to the questionnaires subjects may be asked to voluntary participate in additional procedures, as organized by individual trial sites, which contain the following:

*b) Blood measurement:*

At the baseline visit the study physician will take 4 tubes of venous blood (50 ml in total) from the subject for genetic and epigenetic analyses, measurement of Amyloid-β, Neurofilament light chain (NFL), PBMCs, Metabolomics, Proteomics and miRNA analyses. The total volume of the blood draws will not exceed 50 ml.

*c) MRI:*

A Magnetic Resonance Imaging (MRI) acquisition of the subjects' brain will be performed at baseline to obtain a high-resolution structural image (T1, T2 FLAIR). Furthermore, a resting-state fMRI will be acquired as well as a Diffusion Tensor Imaging (DTI) of the subjects' brain before the psychotherapeutic intervention.

The subjects can participate in the study irrespective of the blood measurements and MRI acquisition. Subjects who fulfill the inclusion criteria and wish to participate in the study but do not agree to the blood draw and/or MRI will be included in the study in any case.

#### 1.11.3.3 Treatment Phase

The intervention of interest is a late life depression specific cognitive behavioural therapy (LLD-CBT). It will be provided over 15 bi-weekly sessions extending over 50 minutes each. The control intervention (supportive unspecific intervention, SUI) will be provided in exactly the same quantity. Both will be delivered in individual face-to-face sessions at the respective trial sites. All study therapists need to have a background of CBT-training, participate in a central two-day workshop of study CBT and study SUI. Before starting with subjects in the study, each study therapist has to demonstrate his/her skills and adherence to study protocols by treating two training subjects. All therapeutic sessions will be filmed. A random selection of at least two treatment sessions per subject will be evaluated by independent raters (group of M. Hautzinger) to monitor adherence to protocol. The raters will be centrally trained and supervised in the application of all outcomes. Regular supervision (at least monthly) by M. Hautzinger and his team to secure adherence to treatment protocols is part of the study protocol.

After being included in the study, the subjects will be randomized to one of the two treatments (LLD-CBT or LLD-SUI) and will begin the first of the successive bi-weekly 50-minute individual face-to-face treatment sessions within one week.

In week 5 (after 7 therapy sessions), following primary and secondary outcomes will be obtained by the blinded rater (T1):

*a) Questionnaires:*

- Geriatric Depression Scale (GDS)
- Quick Inventory of Depressive Symptomatology Clinician-Rated Version (QIDS-C)
- Geriatric Anxiety Inventory (GAI)
- Patient-Reported Outcome in Major Depressive Disorder (PRO-MDD)
- WHO Quality of Life (WHOQOL-OLD and WHOQOL-BREF)

- Health Status (SF 36)
- Insomnia Severity Index (ISI)
- Epworth Sleepiness Scale (ESS)
- REM Sleep Behavior Disorder Screening Questionnaire (RBDSQ)

If applicable to subject:

*b) Blood measurement:*

At T1 the study physician will take 4 tubes of venous blood (50 ml in total) from the subject for epigenetic analyses, measurement of Amyloid-β, NFL, PBMCs, Metabolomics, Proteomics and miRNA analyses. The total volume of the blood draws will not exceed 50 ml.

The outcome assessment at T1 is followed by treatment sessions 8 to 15. Both, the intervention of interest (LLD-CBT) and the control intervention (SUI) will be guided by a detailed manual. The therapist in the study will be centrally trained with regard to both interventions and will also apply both types of intervention.

#### 1.11.3.3.1 LLD-CBT

The LLD-CBT was chosen as the intervention of interest, because

1. CBT in early and midlife is effective in the treatment of depression [9],
2. LLD-CBT is tailored to address LLD-specific psychological factors,
3. the program has shown efficacy on comparison with an active control intervention (SUI) in a pilot study [19].

In that pilot study, the individual face-to-face intervention was more effective than LLD-CBT in a group setting [19]. The number of 15 treatment sessions is required to sufficiently work through the 6 sections of the program. The twice-per-week frequency is optimal to engage the subject in the treatment and it also reflects a real-world scenario of the treatment of subjects with moderate to severe depression. The intervention represents the standard of CBT interventions with regard to number and frequency of session [9]. The LLD-CBT is guided by a manual, which was been developed by the Co-Lead Principal Investigator M. Hautzinger and is based on work by P.M. Lewinsohn, Oregon Research Institute. The manual has been published [16] and has been evaluated in a number of single-centre studies [18, 19]. It is composed of 6 modules which correspond to the basic structure of CBT:

1. Relationship building, history of subject, identification of problems and definition of goals,
2. Provision of the CBT rationale, development of a cognitive-behavioural model of the individual depression, relating interventions to rationale and goals,
3. Stimulation of behavioural activation and day structuring,
4. Development of cognitive techniques and thought control,
5. Building up of social and problem-solving skills,
6. Relapse prevention, crises and emergency plan.

In contrast to CBT programs in young and middle age adults, all modules in the manual are specifically tailored to address the specific characteristic and needs of old and very old people with LLD, which cover specific topics such as experience of loss and physical impairment, but also life review and reminiscence elements, to name a few. Therapists have to cover all modules but have certain flexibility how intensive (number of sessions) each module for an individual subject needs to be worked through. If effective, the mode and scheme of the intervention can be transferred directly into the health care system. In Germany, the CBT intervention would be reimbursed by the health insurance (Richtlinien-Psychotherapie).

#### 1.11.3.3.2 LLD-SUI

In studies on the specific efficacy of a psychotherapy program, it is crucial to apply a control condition of similar quantity and structure as comparator as opposed to a waiting list control group without treatment [37]. The control intervention will be a supportive, unspecific patient-centred, but manual based intervention (SUI) [16] of equivalent quantity as the CBT (15 individual sessions, twice weekly). The therapist will act as an empathic

listener, who will not guide the therapy, but only support the subject in him or her self-reflection and expression of emotions. Educational components with regard to depression are allowed. The therapist will be trained and will be the same person, who is delivering CBT to avoid systematic effects of the therapist. This control intervention has been successfully applied in the pilot study of this multicentre trial [19].

##### 1.11.3.3.3 Allocation to Interventions

After the baseline visit, the subjects will be randomized to either of the two treatment arms (1:1 randomization) according to permuted blocks of varying length. Randomization will be stratified by site in order to prevent unbalanced allocation of the intervention of interest and the control intervention to sites. It will be implemented as a central 24-7 Internet service operated by local, authorized study staff. Treatment assignments will be displayed on screen and delivered by e-mail. The randomization service will be maintained by the IMSB, University of Cologne.

#### 1.11.3.4 Post-treatment phase

End-of-treatment primary and secondary outcomes (T2) will be gained in week 10 after completion of all 15 therapy sessions. The following outcomes will be obtained by the blinded rater at the end of treatment:

*a) Questionnaires:*

- Geriatric Depression Scale (GDS)
- Quick Inventory of Depressive Symptomatology Clinician-Rated Version (QIDS-C)
- Geriatric Anxiety Inventory (GAI)
- Patient-Reported Outcome in Major Depressive Disorder (PRO-MDD)
- WHO Quality of Life (WHOQOL-OLD and WHOQOL-BREF)
- Health Status (SF 36)
- Insomnia Severity Index (ISI)
- Epworth Sleepiness Scale (ESS)
- REM Sleep Behavior Disorder Screening Questionnaire (RBDSQ)

A qualitative interview will be performed at the end of the treatment period to assess individuals' needs and goals met and not met by the intervention.

If applicable to subject:

*b) Blood measurement:*

After completion of the treatment (T2) venous blood samples will be obtained from the subject for epigenetic analyses, measurement of Amyloid-β, NFL, PBMCs, Metabolomics, Proteomics and miRNA analyses. The total volume of the blood draws will not exceed 50 ml.

*c) MRI:*

A Magnetic Resonance Imaging (MRI) acquisition of the subjects' brain will be performed at T2 to obtain a high-resolution structural image (T1 and T2 FLAIR) of the subjects' brain after the psychotherapeutic intervention. Furthermore, a resting-state fMRI will be acquired as well as Diffusion Tensor Imaging (DTI) of the subjects' brain at end-of-treatment (T2).

#### 1.11.3.5 Final examination

6 months after randomization the final follow-up visit (T3) will be performed. Following primary and secondary outcomes will be obtained at the follow-up visit:

*a) Questionnaires:*

- Geriatric Depression Scale (GDS)
- Quick Inventory of Depressive Symptomatology Clinician-Rated Version (QIDS-C)

- Geriatric Anxiety Inventory (GAI)
- Patient-Reported Outcome in Major Depressive Disorder (PRO-MDD)
- WHO Quality of Life (WHOQOL-OLD and WHOQOL-BREF)
- Health Status (SF 36)
- Insomnia Severity Index (ISI)
- Epworth Sleepiness Scale (ESS)
- REM Sleep Behavior Disorder Screening Questionnaire (RBDSQ)
- Consortium to Establish a Registry for Alzheimer's Disease neuropsychological battery (CERADplus)
- Subtests of the Neuropsychological Assessment Battery (NAB) Screening Module (Executive Funtions: Maze)
- Longitudinal Interval Follow-up Evaluation (LIFE)

Furthermore, the history of cognition (subjective cognitive decline) will be assessed at the follow-up visit. A qualitative interview will be performed at the follow-up visit to obtain the medical history during the period from end-of-treatment (T2) to the follow-up visit (T3). This assessment includes but is not limited to the medication history, psychological/psychotherapeutic interventions, other psychiatric interventions (e.g. ECT, TMS, tDCS) and psychosocial stressors in the time interval from end-of-treatment (T2) to follow-up (T3).

If applicable to subject:

b) *Blood measurement:*

At the follow-up visit venous blood samples will be obtained from the subject for epigenetic analyses, measurement of Amyloid-β, NFL, PBMCs, Metabolomics, Proteomics and miRNA analyses. The total volume of the blood draws will not exceed 50 ml.

*c) MRI:*

A Magnetic Resonance Imaging (MRI) acquisition of the subjects' brain will be performed during the follow-up visit to obtain a high-resolution structural image (T1 and T2 FLAIR) of the subjects' brain. Furthermore, a resting-state fMRI will be acquired as well as Diffusion Tensor Imaging (DTI) of the subjects' brain at follow-up (T3).

#### 1.11.3.6 Examination hierarchy and time windows

The screening and the baseline outcome assessment T0 can be performed in one combined visit. In case of separation of screening and baseline assessment, they have to be administered within the same week. This may be necessary for relief of the subjects.

The first treatment session has to be completed within one week after the screening visit. The 15 successive treatment sessions in both groups (LLD-CBT and LLD-SUI) have to be performed in a bi-weekly manner of 50-minute individual face-to-face treatment sessions. If a treatment session can not be proceeded (e.g. sickness of a subject or therapist) it has to be rectified and the session has to be completed within the following week.

The T1 outcome assessment will be performed by the rater in week 5. The outcome assessment T2 at the end of treatment will be carried out in week 10. The follow-up assessment T3 will be performed 6 months after the initial randomization of the subject.

### 1.11.4 Trial conditions and restrictions

#### 1.11.4.1 Medical surveillance

The enrollment into the trial at the screening visit will be performed by the study psychologist or physician. The study physician will perform the venous blood draw from a peripheral vein and be present during the MRI, if applicable. At all trial sites a trial physician and other medically trained employees will be present at the unit.

During all phases of the trial, i.e. from screening until final examination, the subjects can contact medically trained employees at the trial centers and by phone.

#### 1.11.4.2 Precaution and emergencies

It should be stressed that this study does not include risk or harm for the subject. CBT and the control intervention are supportive and goal directed, which limits the risk to a minimum. Neither the intervention of interest nor the control intervention is associated with a significant risk for the subjects. In contrast, it is most likely that both interventions will be of benefit for the subjects (the superiority of LLD-CBT with regard to this benefit is being tested in this trial). None will confront the subjects with negative contents, such a reactivation of trauma as an example. In contrary, both interventions are supportive and aim at stabilizing the mental status of the subjects. The assessments of outcome are also not associated with risk. The Investigators will ensure that the trial is conducted in complete conformance with the principles of Good Clinical Practice (GCP) and the Declaration of Helsinki. Still, adverse events and serious adverse advents will be recorded at every visit and will be reported to a data safety and monitoring board (DSMB) on a continuous basis. For any signs or symptoms or adverse events, a causal or symptomatic treatment according to standard medical practice will be performed, if deemed necessary by the Investigator.

#### 1.11.4.3 General restrictions

During the study, including the follow-up visit, subjects are not allowed to participate in any other clinical trial. They are also not allowed to gain additional inpatient or outpatient psychological/psychotherapeutic treatment throughout the treatment period. Furthermore, subjects must be free of antidepressive medication or have a stable antidepressive pharmacological treatment during the 8-week intervention. The scheduled, regular use of Benzodiazepines (not PRN) is not allowed during the study.

## 1.12 TRIAL VARIABLES

### 1.12.1 Demography

The demographic data to be collected for this trial include i.a.: sex, age, marital and family status, family history, housing situation, education, occupation, nationality/ethnicity, psychiatric and psychotherapeutic history and present treatment, medication, somatic symptoms and diseases, weight, height, BMI, level of physical activity.

### 1.12.2 Adverse events

#### 1.12.2.1 Definition of adverse events

An adverse event (AE) is any untoward medical occurrence in a patient or clinical investigation subject which does not necessarily have a causal relationship with this treatment. An AE can therefore be any unfavorable and unintended sign, symptom, or disease temporally associated with the treatment, whether or not considered related to the treatment.

In this study adverse events are defined and assessed as:

1. Worsening of symptoms e.g. generalisation of symptoms,
2. Occurrence of new symptoms,
3. Occurrence of passive suicidal thoughts,
4. Occurrence of active suicidal intentions or plans,
5. Occurrence of problems in the patient-therapist relationship,
6. Private problems,
7. Occupational problems,
8. Further adverse events (e.g. Influenza, fractures etc.).

### 1.12.2.2 Definition of serious adverse events

A serious adverse event (SAE) is any untoward medical occurrence that at any dose:

1. Results in death,
2. Is life-threatening*,
3. Requires inpatient hospitalization or prolongation of existing hospitalization,
4. Results in persistent or significant disability / incapacity,
5. Is a suicide attempt.

*NOTE: The term 'life-threatening' in the definition of 'serious' refers to an event in which the subject was at risk of death at the time of the event; it does not refer to an event, which hypothetically might have caused death if it was more severe.

An elective hospital admission will not be considered a serious adverse event.

### 1.12.2.3 Definition of intensity of adverse events

The following categories will be used for the rating of the intensity of adverse events:

**Table 3:** Intensity of adverse events

| | |
|---|---|
| Unclear | inadequate data, that are not sufficient for assessment |
| Mild | Causing no limitation of usual activities; the subject may experience slight discomfort |
| Moderate | Causing some limitation of usual activities; the subject may experience annoying discomfort |
| Severe | Causing inability to carry out usual activities; the subject may experience intolerable discomfort or pain |

### 1.12.2.4 Definition of serious adverse event and adverse event causality

A determination of the relationship between an SAE or AE and the treatment must be made after due consideration by the Investigator for each SAE or AE at every visit.

The assessment of the relationship of an SAE or AE to the administration of treatment is a clinical decision based on all available information at the time of and after the occurrence of the event.

The factors which may be considered when evaluating the relationship of an SAE or AE to the treatment include: time from exposure to treatment until onset of the event; recovery or improvement on discontinuation of treatment; availability of alternative explanations such as underlying or intercurrent diseases; concomitant medications or treatments; known response pattern for this treatment; recurrence on reintroduction of treatment.

The relationship of serious adverse events and adverse events to the treatment will be assessed according to the following categories:

- "definite": A clinical event with a reasonable temporal relationship to the administration of the treatment. AE cannot be explained by concomitant diseases or other drugs or chemicals. AE must be phenomenologically unambiguous.
- "probable": A clinical event in a reasonable time sequence to administration of the treatment. AE is unlikely to be attributed to concurrent disease or other drugs or chemicals.
- "possible": A clinical event in a reasonable time sequence to administration of the treatment, but AE could also be explained by concurrent diseases or other drugs or chemicals.

- “unlikely“: A clinical event with a weak temporal relationship to administration of the treatment. Other factors, such as the underlying, concomitant diseases, concomitant medication, are plausible to have contributed to the event.
- “not related“: No relationship to administration of treatment, i.e., there is a clear alternative explanation, an unreasonable temporal relationship between the drug and the event, or relationship is otherwise not plausible.
- “not determinable”: inadequate data, that are not sufficient for assessment.

#### 1.12.2.5 Definition of measures in case of serious adverse event and adverse event

The measures taken in case of AEs will be described according to the following 4 categories:

1. None / treatment continued,
2. Treatment interrupted,
3. Treatment discontinued,
4. Other

The measures taken in case of SAEs will be described according to the following 8 categories:

1. None / treatment continued,
2. Inpatient hospitalization,
3. Non-pharmacological measures,
4. Pharmacological measures,
5. Treatment interrupted,
6. Treatment discontinued,
7. Other.

#### 1.12.2.6 Definition of serious adverse event and adverse event outcome

The outcome of AEs will be described according to the following 5 categories:

1. Resolved,
2. Resolving/solution in process,
3. Not Resolved,
4. Resolved with sequelae,
5. Unknown.

The outcome of SAEs will be described according to the following 6 categories:

1. Resolved,
2. Resolving/solution in process,
3. Not Resolved,
4. Resolved with sequelae,
5. Fatal,
6. Unknown.

#### 1.12.2.7 Suicidality

Suicidality will be assessed at the screening/baseline visit and every following visit during assessment of serious adverse events and adverse events (see section 13.2.1.). Suicidality assessment will include the occurrence of passive suicidal thoughts, active suicidal intentions or plans and suicide attempt since the last visit. The Investigator has to determine the intensity, risk level, causality (study-related or not), measures and outcome.

A determination of the relationship between suicidality and the treatment must be made after due consideration by the Investigator. The assessment of the relationship of suicidality to the administration of treatment is a clinical decision based on all available information at the time of and after the occurrence of the event.

The factors which may be considered when evaluating the relationship of suicidality to the treatment include: time from exposure to treatment until onset of suicidality; recovery or improvement on discontinuation of treatment; availability of alternative explanations such as underlying or intercurrent diseases; concomitant medications or treatments; known response pattern for this treatment; recurrence on reintroduction of treatment.

In case of suicidality (see section 13.2.1. and 13.2.2.) the Investigator has to determine the risk level:

**Table 4:** Suicidality risk level

| | |
|---|---|
| Low risk: | The patient has passive suicidal ideations but no suicidal intentions, plans or active wish to commit suicide. The Investigator/study personnel should pay attention to suicidality and assess the change of status carefully at every visit. The patient must be informed about help services and emergency plans in case of worsening of symptoms. |
| Increased risk: | The patient has suicidal intentions or plans but no active wish to commit suicide. This is an urgent situation. The Investigator/study personnel have to ensure that the patient is being seen by a psychiatrist within 24 hours. The patient must be informed about help services and emergency plans in case of further worsening of symptoms. |
| Acute risk: | The patient has active suicidal intentions or plans. Self-control or external social support might be lacking (e.g. family, friends) or unclear. The study personnel must immediately initiate the admission of the patient to a psychiatric inpatient unit. |

#### 1.12.2.8 Adverse event recording and reporting

The risk of CBT and of the control intervention is minimal. Theoretically psychotherapeutic intervention can be harmful by inducing worsening of symptoms. CBT and the control intervention, however, are supportive and goal directed, which limits the risk to a minimum. The interventions will be provided by experienced psychotherapist, which will also limit the risk of harm. Still, adverse events and serious adverse advents will be recorded at every visit and captured in the eCRF. A data safety and monitoring board (DSMB) will be established, to which safety data will be reported on a continuous basis.

#### 1.12.2.9 Annual safety report

Not applicable.

## 1.13 DOCUMENTATION AND ARCHIVING OF TRIAL DATA

### 1.13.1 Data Management

The data management infrastructure and staff will be supplied by the CTCC. The CTCC will use the validated commercial system TrialMaster. The database is integrated into a general IT infrastructure and safety concept with a firewall and backup system. The data are backed up daily. The trial software has a user and role concept that can be adjusted on a trial-specific basis ensuring that only authorized persons can enter or change data. All changes made to the data are documented in an audit tail. The trial database will be developed and validated before data entry based on standard operating procedures at the CTCC. Data will be entered online at the trial sites via the internet. Automatic plausibility checks are run during data entry. The CTCC data management department will conduct further checks. Queries will be raised in case of missing or implausible data entries. These electronic queries have to be answered by the trial site without unreasonable delay. Details are described in a data management manual. All tasks of CTCC will be performed according to standard operation procedures of CTCC.

After completion and cleaning of data, the database is locked and the data exported for statistical analysis. After the end of trial data management will provide the eCRF data in a format like PDF for data archiving.

### 1.13.2 Electronic Case Report Form (eCRF)

An electronic Case Report Form (eCRF) will be used to record all of the information required by the protocol to be reported on each trial subject. The CTCC Data management will develop the trial eCRF together with the clinical project manager, the principal coordinating investigator and the IMSB, to assure practicability and validity of the eCRF. Access to the eCRF will be provided to the Investigator site team as required after passing an online training (eLearning).

In case of unavailability of eCRF due to IT related problems with database access, each site gets a PDF of a casebook for paper-based documentation of subject's data. This data has to be transferred to the eCRF, when the database is available again.

### 1.13.3 Source data

Source data is all information in original records and certified copies of original records of clinical findings, observations, or other activities in a clinical trial necessary for the reconstruction and evaluation of the trial. Source data are contained in source documents which comprise clinical documentation, data and records (e.g. hospital records, clinical and office charts, laboratory notes, memoranda, evaluation checklists, pharmacy dispensing records, recorded data from automated instruments and data and records arising from other departments such as the pharmacy, laboratory and medico-technical departments).

Clinical documentation relevant to the trial includes all records in any form (including, but not limited to, written, electronic, magnetic and optical records, and scans) that describe or record the methods, conduct and/or results of the trial, the factors affecting the trial and the actions taken.

All clinical documentation and data arising from the trial are to be kept by the Investigator/ trial site.

There should be documentation in the subject's clinical file of any recordings or data specifically obtained for the trial (e.g. questionnaires).

#### 1.13.3.1 Coding

Medical history and adverse events will be coded using MedDRA. The version most currently implemented at CTCC will be used.

### 1.13.4 Data archiving

The trial master file, the eCRF data files and other material supplied for the performance will be archived for at least 10 years by the Sponsor/Lead Principal Investigator in order to fulfil the requirements. Trial subject identification lists and source data at each trial site will be stored separately from Investigator Site File for at least 10 years.

## 1.14 STATISTICAL METHODS

### 1.14.1 Determination of sample size

In a pilot trial [19] the experimental group (n=27) improved from 19.26±3.92 to 10.67±6.42 (mean±SD) and the control (n=25) from 20.68±3.96 to 14.92±7.58 on the GDS. Thus, assuming correlation of 0.5 between measurements (pre to 6 months post randomization), a standardized difference of about 0.52 (≈2.83/5.47) was observed between groups. Our multicentre trial should be powerful enough to detect a clinically relevant difference of 2.5 GDS points (d=0.4). Thus, the two-sample t-test requires 99 subjects per group to reach 80% power at two-sided significance level 5%. Accounting for up to 20% attrition and stratification, 124 (≈99/0.8) subjects need to

get randomized per group, i.e. 248 subjects in total. Adjusting for baseline in a mixed model for repeated measures (MMRM) approach is likely to further increase the statistical power (see below). Nota bene, due to stratification by therapist, the precision of the treatment effect is not affected by between-therapist variation [35].

### 1.14.2 Randomization

This trial is a controlled trial with randomization. Randomization is the process of assigning trial subjects to treatment or control groups using an element of chance to determine the assignments in order to reduce bias. Subjects are assigned to treatment arms (1:1) by means of the central 24-7 internet randomization service ALEA. The randomization will be stratified by centre. The allocation sequence is made from permuted blocks of varying length, where block size is unknown to the investigators. In the rare case of unavailability of the service, a fax-based fallback procedure is used. When all (100%) of the data have been cleaned and the database locked, the biostasticians will be given access to the randomization code.

### 1.14.3 Analysis of the trial

There are three different analysis sets:

**Full analysis set (FAS)**

The primary (full) analysis set (FAS) is derived from the intention-to-treat principle (all subjects randomized with a valid baseline assessment and at least one valid follow-up outcome assessment).

**Per-protocol-set (PPS)**

The per-protocol-set is defined as a subset of the FAS population (all subjects without major protocol violations and at least 9 sessions in one of the therapeutic intervention as scheduled in the trial protocol).

**Safety Population**

All subjects with at least one treatment.

**Primary Endpoint**

The primary outcome measure „change in GDS from baseline to end-of-treatment“ is evaluated by a mixed model for repeated measures (MMRM) with fixed effects baseline, therapist, group, time and the interaction group*time (ARH1-structured covariance matrix over time) with corresponding marginal means and contrast tests. Data from low recruiting therapists (i.e. <4 subjects) are pooled by study centre. The potential clustering of observations of participants by therapist or centre will be investigated by multilevel modelling [5]. Since mixed models can be expected to yield valid results only in case of missingness-at-random, multiple imputation approaches are taken to assess the robustness of the results. Specifically, missing values due to death, illness or chance are separately imputed assuming mixtures of missingness-not-at-random patterns. Imputation data sets are post-processed by multiplication with factors and addition of offsets (tipping point analysis) [8]. Proxy measures are taken into account to ameliorate the effects of attrition.

**Secondary Enpoints**

Secondary outcomes (i.e. further time points and measures) are analyzed along the same lines, i.e. using mixed modeling (or GEEs). Time-to-event (e.g. drop-out or survival) distributions are summarized by the Kaplan-Meier method and compared by the (stratified) log-rank test. Analysis of the set of subjects essentially observed and treated per protocol (PP) is supportive; similarly the compliers' average causal effect (CACE) is calculated [11]. Subgroup analyses are done by study site and gender (expected male to female ratio 1:2); interaction with treatment is investigated.

Quantitative outcomes are summarized by mean, standard deviation and percentiles (i.e. 0, 25, 50, 75, 100), qualitative outcomes by count (percentage). All the details, particularly regarding how to deal with missing data and attrition, are documented in a statistical analysis plan to be finalized before start of enrollment.

#### 1.14.3.1 Analysis of demographic data and other data of interest

Demographic data will be described overall with respect to all relevant populations. For age, weight, height, and body mass index descriptive statistics (n, mean, standard deviation, minimum, Q1, median, Q3, maximum) will be provided. For the categorical variables (e.g. ethnic group, gender) absolute and relative frequencies will be tabulated.

#### 1.14.3.2 Safety analysis

All safety and tolerability parameters (adverse events, serious adverse events) will be listed by subject and treatment group. All analyses will be performed for the safety population and stratified by treatment group.

## 1.15 DATA QUALITY ASSURANCE

### 1.15.1 Monitoring

Monitoring is the process of overseeing the progress of a clinical trial, and of ensuring that the rights and well-being of subjects are protected; that the trial is conducted, recorded, and reported in accordance with the protocol, SOPs, GCP and applicable regulatory requirements; and that the trial data are accurate, complete and verifiable from source data.

Monitoring is done in a risk-adapted manner. The monitoring strategy will be outlined in a Monitoring Plan.

Monitoring of the trial site will be performed by CRAs appointed by CTCC according to the Monitoring Plan.

### 1.15.2 Quality system, audit and inspection

#### 1.15.2.1 Quality system

CTCC is responsible for implementing and maintaining quality assurance and quality control systems with written SOPs. The Sponsor is responsible for ensuring that all parties involved with the trial agree to direct access to all trial related sites, source data, documents and reports for the purposes of monitoring and auditing by the Sponsor Quality Assurance unit and CTCC as well as inspections by ECs and the BMBF.

#### 1.15.2.2 Audit/Inspections

As part of quality assurance, the sponsor has the right to audit the trial sites and any other institutions involved in the trial. The aim of an audit is to verify the validity, accuracy and completeness of data, to establish the credibility of the clinical trial, and to check whether the trial subject's rights and safety are being maintained. The sponsor may assign these activities to persons otherwise not involved in the trial (auditors). These persons are allowed to get access to all trial documentation (especially the trial protocol, electronic case report forms, trial subjects' medical records and trial-related correspondence).

The sponsor and all trial sites involved will support auditors and inspections by ECs and the BMBF at all times and allow the persons charged with these duties to have access to the necessary original documentation. All persons conducting audits undertake to keep all trial subject data and other trial data confidential.

## 1.16 GENERAL CONDITIONS AND AGREEMENTS

### 1.16.1 Amendment of protocol

To ensure comparable conditions for all study subjects and a proper future evaluation, no change of the agreed study conditions outlined in the study protocol is intended. Exceptional changes that become necessary during the study sequence are implemented by the Lead Principal Investigator and the Co-Lead Principal Investigator of the

study in coordination with the CTCC and other persons who are significantly involved in the compilation of the protocol.

Changes to the protocol during the trial will be documented as amendments and become an integral component of the study protocol. Any change of this kind must be made in writing including the specification of the reasons. The amendment must be added to every circulating copy of the study protocol at the trial sites.

Depending on the contents of the amendment and local legal requirements, the amendment will be submitted to the relevant ECs.

The Investigator must not implement any deviation from, or changes of the protocol, without agreement by the Lead Principal Investigator and prior review and documented approval/favourable opinion of the appropriate EC, except where necessary to eliminate an immediate hazard to the subjects.

If an amendment substantially alters the trial design, increases the potential risk to the subjects or affects the treatment of the subject, then the information sheet must be revised and submitted to the relevant EC for review and approval. When a subject is currently undergoing trial procedures and is affected by the amendment, then the subject must be asked to consent again using the new information sheet. The new information sheet must be used to obtain consent from new subjects before enrollment.

In case an addition to the study protocol is required for purely administrative or technical changes of the study protocol which have no bearing on the subject's health-related interests, only the notification of the competent ethics committee is required.

### 1.16.2 Publication policy

The results of this study will be presented on national and international scientific conferences on psychiatry, psychotherapy and psychology and will be published in peer reviewed journals. Since this will be the largest study on CBT in LLD, results will most likely enter the national guideline on the treatment of unipolar depression (Nationale Versorgungsleitlinie unipolare Depression) and the international guidelines. Publications will be prepared under the primary authorship of the site in charge of managing the study (University of Cologne, Department of Psychiatry and Psychotherapy) once the biometric evaluation and the clinical biostatistical evaluation report is complete.

If any investigator would like to publish or present partial or complete site data from this trial, they must obtain a prior written permission from the Steering Board of the trial. The Steering Board consists of the Lead Principal Investigator of the trial Prof. Dr. med. F. Jessen, the Co-Lead Principal Investigator Prof. Dr. M. Hautzinger, the Principal Investigators at the other 5 trial sites and the clinical project manager. The Steering Board meets regularly and defines the publication policy. In case the Steering Board gives its consent, the investigator agrees to provide the Steering Board with the planned publication for its review in due time at least 45 days prior to its submission. All co-authors are given the opportunity to provide feedback within a reasonable period before the manuscript/abstract is submitted for publication. Objections from the Steering Board have to be taken into account. In case of any discrepancies between the provisions contained herein and the terms and conditions set forth in the contract with the investigational sites and the investigators the provisions of the protocol shall prevail.
The results will be additionally communicated by the subject advocacy organization Deutsche Depressionsliga e.V. and Stiftung Deutsche-Depressionshilfe. To ensure data management, maintenance and long-term accessibility for future reuse of the results of the trial (also by third parties), all data will be archived at the University of Cologne in anonymized form using standard data formats. On request, researchers may have access to the data and results of the trial after obtaining a written permission from the Steering Board. Data will have to be fully compatible with the individual privacy of trial participants and data protection rules as defined by regulatory requirements and the patient informed consent form. The Steering Board of the study will decide on data request by third parties.

### 1.16.3 Interim reports

No interim analysis is planned, so interim reports will only be provided in case of premature termination of the study.

### 1.16.4 Final report

The ethics committee will be informed within 90 days that the trial has officially ended. Within one year after completion of the trial, the ethics committee will be supplied with a summary of the final report or an adequate publication on the clinical trial containing the principle results. A final report to the funding body (BMBF) will be provided according to the funding requirements.

### 1.16.5 Confidentiality of trial results

The results of this trial are confidential and are not to be transmitted to a third party in any form or fashion. All persons involved in the trial are bound by this confidentiality clause.

## 1.17 REFERENCES


1. Almeida OP, Hankey GJ, Yeap BB et al. Depression as a modifiable factor to decrease the risk of dementia. Transl Psychiatry 2017; 7(5):1-6.

2. Amick HR, Gartlehner G, Gaynes BN, et al. Comparative benefits and harms of second generation antidepressants and cognitive behavioral therapies in initial treatment of major depressive disorder: systematic review and meta-analysis. BMJ. 2015:h6019. doi:10.1136/bmj.h6019.

3. Arroll B, Elley CR, Fishman T, et al. Antidepressants versus placebo for depression in primary care. Cochrane Database Syst Rev. 2009;(3):477-485. doi:10.1002/14651858.CD007954.

4. Aziz R, Steffens DC. What Are the Causes of Late-Life Depression? Psychiatr Clin North Am 2013; 36(4): 497–516.

5. Boutron I, Moher D, Altman DG, Schulz KF, Ravaud P. Extending the CONSORT statement to randomized trials of nonpharmacologic treatment: Explanation and elaboration. Ann Intern Med. 2008;148:295-309. doi:10.7326/0003-4819-148-4-200802190-00008.

6. Buechtemann D, Luppa M, Bramesfeld A, Riedel-Heller S. Incidence of late-life depression: A systematic review. J Affect Disord. 2012;142(1-3):172-179. doi:10.1016/j.jad.2012.05.010.

7. Busch MA, Maske UE, Ryl L, Schlack R, Hapke U. Prävalenz von depressiver Symptomatik und diagnostizierter Depression bei Erwachsenen in Deutschland: Ergebnisse der Studie zur Gesundheit Erwachsener in Deutschland (DEGS1). Bundesgesundheitsblatt - Gesundheitsforsch - Gesundheitsschutz. 2013;56(5-6):733-739. doi:10.1007/s00103-013-1688-3.

8. Buuren S van, Groothuis-Oudshoorn K. mice: Multivariate Imputation by Chained Equations in R. J Stat Softw. 2011;45(3). doi:10.18637/jss.v045.i03.

9. DGPPN, BÄK, KBV, AWMF, AkdÄ, BPtK, BApK, DAGSHG, DEGAM, DGPM, DGPs, DGRW (Hrsg.) für die Leitliniengruppe Unipolare Depression*. S3-Leitlinie/Nationale VersorgungsLeitlinie Unipolare Depression – Langfassung. 2.Auflage. Version 4. 2015. Available from: www.depression.versorgungsleitlinien.de; [cited: 02.11.2017]; DOI: 10.6101/AZQ/000329

10. Diniz B, Reynolds III C, Butters M, et al. The effect of gender, age, and symptom severity in late-life depression on the risk of all-cause mortality: The Bambuí Cohort Study of Aging. Depress Anxiety. 2014;31(9):787-795. doi:10.1038/ajh.2011.80.Arterial.

11. Dunn G, Maracy M, Dowrick C, et al. Estimating psychological treatment effects from a randomised controlled trial with both non-compliance and loss to follow-up. Br J Psychiatry. 2003;183(4):323-331.

12. Fiske A, Wetherell JL, Gatz M. Depression in Older Adults. Annu Rev Clin Psychol. 2009;5(1):363-389. doi:10.1146/annurev.clinpsy.032408.153621.

13. Gould RL, Coulson MC, Howard RJ. Cognitive behavioral therapy for depression in older people: A meta-analysis and meta-regression of randomized controlled trials. J Am Geriatr Soc. 2012;60(10):1817-1830. doi:10.1111/j.1532-5415.2012.04166.x.

14. Gühne U, Luppa M, König HH, Hautzinger M, Riedel-Heller S. Ist Psychotherapie bei depressiven Erkrankungen im Alter wirksam?: Ein systematischer Überblick. Psychiatr Prax. 2014;41:415-423. doi:10.1055/s-0034-1370113.

15. Haigh EAP, Bogucki OE, Sigmon ST, Blazer DG. Depression Among Older Adults: A 20-Year Update on Five Common Myths and Misconceptions. Am J Geriatr Psychiatry. 2017:1-16. doi:10.1016/j.jagp.2017.06.011.

16. Hautzinger M. Depression Im Alter. Psychotherapeutische Behandlung Für Das Einzel- Und Gruppensetting. 2nd ed. Weinheim: Beltz; 2016.

17. Hautzinger M, Dykierek P, Fellgiebel A, Hüll M. Psychotherapeutische Ansätze bei der Behandlung älterer Menschen. Nervenarzt. 2017: published online: 12 Sep 2017. doi:10.1007/s00115-017-0404-1.

18. Hautzinger M, Welz S. Kognitive Verhaltenstherapie bei Depressionen im Alter: Ergebnisse einer kontrollierten Vergleichsstudie unter ambulanten Bedingungen an Depressionen mittleren Schweregrads. Z Gerontol Geriatr. 2004;37(6):427-435. doi:10.1007/s00391-004-0262-x.

19. Hautzinger M, Welz S. Kurz- und längerfristige Wirksamkeit psychologischer Interventionen bei Depressionen im Alter. Z Klin Psychol Psychot. 2008;37:52-60.

20. Hautzinger M. Kognitive Verhaltenstherapie Depressiver Störungen Im Alter. Psychotherapie. 2000;5(1):122-128.

21. Holman AJ, Serfaty MA, Leurent BE, King MB. Cost-effectiveness of cognitive behaviour therapy versus talking and usual care for depressed older people in primary care. BMC Health Serv Res. 2011;11(1):33. doi:10.1186/1472-6963-11-33.

22. Jonsson U, Bertilsson G, Allard P, et al. Psychological treatment of depression in people aged 65 years and over: A systematic review of efficacy, safety, and cost-effectiveness. PLoS One. 2016;11(8):1-20. doi:10.1371/journal.pone.0160859.

23. Koenig AM, Bhalla RK, Butters MA. Cognitive Functioning and Late-Life Depression. J Int Neuropsychol Soc 2014; 20(5): 461–467.

24. Kok RM, Nolen WA, Heeren TJ. Efficacy of treatment in older depressed patients: A systematic review and meta-analysis of double-blind randomized controlled trials with antidepressants. J Affect Disord. 2012;141(2-3):103-115. doi:10.1016/j.jad.2012.02.036.

25. Kok RM, Reynolds CF. Management of Depression in Older Adults: A Review. JAMA. 2017;317(20):2114. doi:10.1001/jama.2017.5706.

26. Lasch KE, Hassan M, Endicott J, et al. Development and content validity of a patient reported outcomes measure to assess symptoms of major depressive disorder. BMC Psychiatry. 2012;12(1):34. doi:10.1186/1471-244X-12-34.

27. Leonard BE. Major Depression as a Neuroprogressive Prelude to Dementia: What Is the Evidence? Mod Trends Pharmacopsychiatry. 2017; 31: 56-66. doi:https://dx.doi.org/10.1159/000470807.

28. Luppa M, Sikorski C, Luck T, et al. Age- and gender-specific prevalence of depression in latest-life - Systematic review and meta-analysis. J Affect Disord. 2012;136(3):212-221. doi:10.1016/j.jad.2010.11.033.

29. Luppa M, Sikorski C, Motzek T, Konnopka A, König H, Riedel-Heller S. Health Service Utilization and Costs of Depression in Late Life - A Systematic Review. Curr Pharm Des. 2012;18(36):5936-57. doi:10.1055/s-0032-1322056.

30. Luppa M, König H-H, Heider D, et al. Direct costs associated with depressive symptoms in late life: a 4.5-year prospective study. Int Psychogeriatrics. 2013; 25(2):292-302. doi:10.1017/S1041610212001688.

31. Mirza SS, Wolters FJ, Swanson SA et al. 10-year trajectories of depressive symptoms and risk of dementia: a population-based study. Lancet Psychiatry 2016; 3(7): 628-635.

32. Ownby RL, Crocco E, Acevedo A et al. Depression and Risk for Alzheimer Disease. Systematic Review, Meta-analysis, and Metaregression Analysis. Arch Gen Psychiatry 2006; 63: 530-538.

33. Raue PJ, McGovern AR, Kiosses DN, Sirey JA. Advances in Psychotherapy for Depressed Older Adults. Curr Psychiatry Rep. 2017; 19(57):1-9.doi:10.1007/s11920-017-0812-8.

34. Reynolds CF III, Alexopoulos GS, Katz IR, Lebowitz BD. Chronic depression in the elderly: Approaches for prevention. Drugs & Aging. 2001; 18(7):507–514.

35. Roberts C, Roberts SA. Design and analysis of clinical trials with clustering effects due to treatment. Clin Trials. 2005; 2(2):152-162.doi:10.1191/1740774505cn076oa.

36. Serfaty MA, Haworth D, Blanchard M, Buszewicz M, Murad S, King M. Clinical Effectiveness of Individual Cognitive Behavioral Therapy for Depressed Older People in Primary Care. Arch Gen Psychiatry. 2009; 66(12):1332. doi:10.1001/archgenpsychiatry.2009.165.

37. Shinohara K, Honyashiki M, Imai H, Hunot V, Davies P, Churchill R. Behavioural therapies versus other psychological therapies for depression. [Review]. Cochrane database Syst Rev. 2013; 10(10):CD008696. doi:http://dx.doi.org/10.1002/14651858.CD008696.pub2.

38. Singh-Manoux A, Dugravot A, Fournier A et al. Trajectories of Depressive Symptoms Before Diagnosis of Dementia A 28-Year Follow-up Study. JAMA Psychiatry 2017; 74(7): 712-718.

39. Statistisches Bundesamt Gesundheit: Todesursachen in Deutschland 2015. 2015. Im Internet: https://www.destatis.de/DE/Publikationen/Thematisch/Gesundheit/Todesursachen/Todesursachen.html

40. Van Schaik DJF, Klijn AFJ, Van Hout HPJ, et al. Patients' preferences in the treatment of depressive disorder in primary care. Gen Hosp Psychiatry. 2004;26(3):184-189. doi:10.1016/j.genhosppsych.2003.12.001.

41. Volkert J, Schulz H, Härter M et al. The prevalence of mental disorders in older people in Western countries – a meta-analysis. Ageing Res Rev 2013; 12(1): 339-353.

42. Weyerer S, Eifflaender-Gorfer S, Wiese B, et al. Incidence and predictors of depression in non-demented primary care attenders aged 75 years and older: Results from a 3-year follow-up study. Age Ageing. 2013; 42(2):173-180. doi:10.1093/ageing/afs184.

43. Wilson K, Mottram P, Vassilas C. Psychotherapeutic treatments for older depressed people. Cochrane Database Syst Rev. 2008;23(1):CD004853.

44. World Health Organization. The Global Burden of Disease: 2004 update. World Heal Organ. 2004:1-160. doi:10.1038/npp.2011.85.

45. Yesavage JA, Brink TL, Rose TL, et al. Development and Validation of a Geriatric Depression Report Screening Scale: a Preliminary. J Psychiatr Res. 1983; 17(1):37–49. doi:10.1016/0022-3956(82)90033-4.

## 1.18 APPENDIX

### 1.18.1 Planned participating trial sites

1) Department of Psychiatry and Psychotherapy, University Medical Center Cologne (Site Principal Investigator: Prof. Dr. Frank Jessen)
2) Department of Clinical and Developmental Psychology, University of Tuebingen (Site Principal Investigator: Prof. Dr. Martin Hautzinger)
3) Department of Psychiatry and Psychotherapy, University Medical Center Bonn (Site Principal Investigator: Prof. Dr. Michael Wagner)
4) Department of Psychiatry on Campus Benjamin Franklin, Charité Berlin (Site Principal Investigator: PD Dr. Oliver Peters, MD)
5) Department of Psychiatry and Psychotherapy, University Medical Center Freiburg (Site Principal Investigator: Prof. Dr. Elisabeth Schramm; Dr. Magnus-Sebastian Vry)
6) Institute of Social Health, Occupational Health and Public Health (ISAP), University of Leipzig (Site Principal Investigator: Prof. Dr. Steffi G. Riedel-Heller)
7) Department of Psychiatry, Central Institute for Mental Health Mannheim (Site Principal Investigator: Prof. Dr. Lutz Frölich, MD)

# 2 Statistical Analysis Plan

## 2.1 Background

### 2.1.1 Trial objective

To test the hypothesis that a 15-session individuallydelivered cognitive behavioural therapy (CBT) specific for late life depression (LLD) is of superior efficacy in reducing symptoms of depression in comparison with a supportive unspecific intervention (SUI) of the same quantity in patients with LLD.

### 2.1.2 Trial design

This is a randomized, multi-center, single-blind (observer-blinded), active-controlled, parallel group trial. The planned number of subjects is 300 subjects assessed for eligibility and 248 allocated to the trial. The planned number of subjects per treatment (LLD-CBT, LLD-SUI) is 124.

### 2.1.3 Treatments

Experimental group: A manual-based, individual, 15-session, twice weekly, outpatient CBT specifically designed for patients with late life depression (LLD-CBT) versus

Control group: A manual-based 15-session, twice weekly, outpatient standard treatment containing supportive and educational elements (supportive unspecific intervention, LLD-SUI)

### 2.1.4 Blinding

This is a single-blind (observer-blinded) trial. The primary and secondary outcomes will be obtained at baseline, in week 5 after 7 treatment sessions, in week 10 at the end of treatment and at follow-up (6 months after randomization) by the rater, who will be blinded to the treatment arm allocation.

### 2.1.5 Randomisation

After the baseline visit, the subjects are assigned to treatment arms (1:1) by means of the central 24-7 internet randomization service ALEA. The randomization will be stratified by centre. The allocation sequence is made from permuted blocks of varying length, where block size is unknown to the investigators.

Figure 1: Trial Flow Chart

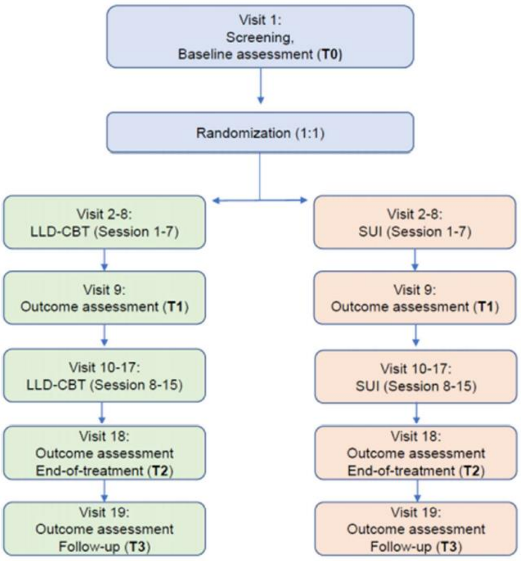


Figure 2: Trial Schedule

| Procedure | TRIAL SCHEDULE | | | | | | | | | | | | | | | | | | | |
|---|---|---|---|---|---|---|---|---|---|---|---|---|---|---|---|---|---|---|---|---|
| | Screening | Baseline | Treatment | | | | | | | | | | | | | | | | End of treatment | Follow-up |
| | SCR | T0 | | | | | | | | T1 | | | | | | | | | T2 | T3 |
| **Visit** | 1 | 1 | 2 | 3 | 4 | 5 | 6 | 7 | 8 | 9 | 10 | 11 | 12 | 13 | 14 | 15 | 16 | 17 | 18 | 19 |
| Week (W)<br>Month (M) | W1 | W1 | W1 | W2 | W2 | W3 | W3 | W4 | W4 | W5 | W5 | W5 | W6 | W6 | W7 | W7 | W8 | W8 | W10 | M6 |
| Written informed consent obtained | • | | | | | | | | | | | | | | | | | | | |
| Evaluation of in-/ exclusion criteria | • | | | | | | | | | | | | | | | | | | | |
| Demographic data | | • | | | | | | | | | | | | | | | | | | |
| Concomitant diseases/ medication [a)] | • | | | | | | | | | | | | | | | | | | | |
| Medical history | | • | | | | | | | | | | | | | | | | | | |
| Structured clinical interview (MINI 7.0.2) | • | | | | | | | | | | | | | | | | | | | |
| GDS | • | | | | | | | | | • | | | | | | | | | • | • |
| QIDS-C | • | | | | | | | | | • | | | | | | | | | • | • |
| MMST | • | | | | | | | | | | | | | | | | | | | |
| GAI | | • | | | | | | | | • | | | | | | | | | • | • |
| PRO-MDD | | • | | | | | | | | • | | | | | | | | | • | • |
| WHO-QOL BREF & OLD | | • | | | | | | | | • | | | | | | | | | • | • |
| SF36 | | • | | | | | | | | • | | | | | | | | | • | • |
| CTQ-SF | | • | | | | | | | | | | | | | | | | | | |
| BFI-10 | | • | | | | | | | | | | | | | | | | | | |
| ISI/ESS/RBDSQ | | • | | | | | | | | • | | | | | | | | | • | • |
| CERADplus | | • | | | | | | | | | | | | | | | | | | • |
| NAB subtest maze | | • | | | | | | | | | | | | | | | | | | • |
| LIFE | | | | | | | | | | | | | | | | | | | | • |
| Treatment (CBT or SUI) | | | • | • | • | • | • | • | • | | • | • | • | • | • | • | • | • | | |

#### 2.1.6 Timing of Analyses

The final analysis of the primary outcome will be performed after the completion of the last visit of the last patient.

#### 2.1.7 Sample size

In a pilot trial [19] the experimental group (n=27) improved from 19.26±3.92 to 10.67±6.42 (mean±SD) and the control (n=25) from 20.68±3.96 to 14.92±7.58 on the GDS. Thus, assuming correlation of 0.5 between measurements (pre to 6 months post randomization), a standardized difference of about 0.52 (≈2.83/5.47) was observed between groups. Our multicentre trial should be powerful enough to detect a clinically relevant difference of 2.5 GDS points (d=0.4). Thus, the two-sample t-test requires 99 subjects per group to reach 80% power at two-sided significance level 5%. Accounting for up to 20% attrition and stratification, 124 (≈99/0.8) subjects need to get randomized per group, i.e. 248 subjects in total. Adjusting for baseline in a mixed model for repeated measures (MRM) approach is likely to further increase the statistical power (see below).

### 2.2 Analysis populations

#### 2.2.1 Definitions

Full analysis set (FAS)

- The primary (full) analysis set (FAS) is derived from the intention-to-treat principle (all subjects randomized with a valid baseline assessment and at least one valid followup outcome assessment). Per-protocol-set (PPS)
- The per-protocol-set is defined as a subset of the FAS population (all subjects without major protocol violations and at least 9 sessions in one of the therapeutic intervention as scheduled in the trial protocol).

Safety analysis set (SAF)

- All subjects with at least one treatment.

#### 2.2.2 Major protocol violations / Withdrawals

Each subject will be informed that participation is voluntary and withdrawal is possible at any time. Subjects who withdraw or stop treatment prematurely will not be replaced.

Additionally to the withdrawal of subjects, the Investigator can stop the participation of a subject after consideration of the benefit/risk ratio. Other possible reasons for the Investigator to discontinue a subject’s trial treatment or participation include:

- Any relevant deterioration in the health of the subject possibly impacting the participation in the trial, including adverse events, severe medical condition, major surgery, laboratory parameters, vital signs or other safety parameters at the discretion of the Investigator
- Regular use with scheduled dosing of benzodiazepines (NB: not PRN) or change of antidepressive pharmacological treatment during 8-week intervention

- Parallel psychiatric or psychotherapeutic interventions (including but not limited to ECT, TMS, tDCS, VNS) during the treatment period
- Technical reasons (e.g. subject moves away)

## 2.3 Trial centres

Planned participating trial sites:

| | | | |
|---|---|---|---|
| 1 | Cologne, Germany | Department of Psychiatry and Psychotherapy, University Medical Center Cologne | Prof. Dr. Frank Jessen |
| 2 | Tuebingen, Germany | Department of Clinical and Developmental Psychology, University of Tuebingen | Prof. Dr. Martin Hautzinger |
| 3 | Bonn, Germany | Department of Psychiatry and Psychotherapy, University Medical Center Bonn | Prof. Dr. Michael Wagner |
| 4 | Berlin, Germany | Department of Psychiatry on Campus Benjamin Franklin, Charité Berlin | PD Dr. Oliver Peters, MD |
| 5 | Freiburg, Germany | Department of Psychiatry and Psychotherapy, University Medical Center Freiburg | Prof. Dr. Elisabeth Schramm; Dr. MagnusSebastian Vry |
| 6 | Leipzig, Germany | Institute of Social Health, Occupational Health and Public Health (ISAP), University of Leipzig | Prof. Dr. Steffi G. Riedel-Heller |
| 7 | Mannheim, Germany | Department of Psychiatry, Central Institute for Mental Health Mannheim | Prof. Dr. Lutz Frölich, MD |

## 2.4 Analysis variables

### 2.4.1 Demography and baseline characteristics

Demographic variables

- Sex
- Age
- Marital status
- Family status
- Family history
- Housing situation
- Education
- Occupation
- Nationality/ethnicity

Medical variables

- Psychiatric and psychotherapeutic history
- Psychiatric and psychotherapeutic present treatment
- Medication
- Somatic symptoms
- Somatic diseases
- Weight
- Height

- BMI
- Level of physical activity
- Blood measurement o Genetic and epigenetic analyses o Measurement of Amyloid-β and Neurofilament light chain (NFL) o miRNA analyses o Metabolomics and Proteomics o PBMC analyses
- Magnetic Resonance Imaging (MRI) o High-resolution structural image o resting-state fMRI o Diffusion Tensor Imaging (DTI)

### 2.4.2 Primary variable

- Change in Geriatric Depression Scale (GDS) from baseline to end-of-treatment (week 10)

### 2.4.3 Secondary variables

- Change in Geriatric Depression Scale (GDS) from baseline to end of follow-up (month 6)
- Changes from baseline to end-of-treatment and to end of follow-up in:
    - o Quick Inventory of Depressive Symptomatology (QIDS-C) o Geriatric Anxiety Inventory (GAI) o Patient-Reported Outcome in Major Depressive Disorder (PRO-MDD) o Health Status (SF 36) o Insomnia Severity Index (ISI) o Epworth Sleepiness Scale (ESS) o EM Sleep Behavior Disorder Screening Questionnaire (RBDSQ) o Consortium to Establish a Registry for Alzheimer's Disease neuropsychological battery (CERADplus)
    - o Subtest of the Neuropsychological Assessment Battery (NAB)
- Influences of traumatic experiences, and personality traits on the change in GDS will be assessed at baseline by:
    - o Childhood Trauma Questionnaire (CTQ-SF) o Big Five Inventory-10 (BFI-10)
- Longitudinal Interval Follow-up Evaluation (LIFE)

#### 2.4.3.1 Safety/Tolerability

- Adverse events
- Serious adverse events
- Assessment of suicidality

#### 2.4.3.2 Quality of life

- WHO Quality of Life (WHOQOL-OLD, WHOQOL-BREF)

## 2.5 Handling of missing values and outliers

### 2.5.1 Missing values

Missing values will not be substituted; however, see below section 6.3.

### 2.5.2 Outliers

No outlier analysis will be done.

## 2.6 Statistical analyses / methods

### 2.6.1 Patient / Subject Disposition

The number of patients screened will be determined from local documentation. The frequency of screening failures due to specific inclusion and exclusion criteria will be reported. Multiple selections per patient were possible.

Application of the inclusion and exclusion criteria to all included subjects will be verified. Frequencies will be shown in a subject flow diagram. Number of and reasons for drop-outs will be included.

### 2.6.2 Demography and baseline characteristics

Demographic and baseline characteristics will be summarized descriptively by treatment arm for ITT, PPS, and SAF.

Descriptive statistical analyses include: Number of subjects (n), arithmetic mean, standard deviation (SD), median, first quartile (Q1), third quartile (Q3), minimum (min), maximum (max) for quantitative variables and absolute and relative frequencies for qualitative variables.

### 2.6.3 Primary analysis

The primary objective of this study is to test the hypothesis that a 15-session individuallydelivered cognitive behavioural therapy (CBT) specific for late life depression (LLD) is of superior efficacy in reducing symptoms of depression in comparison with a supportive unspecific intervention (SUI) of the same quantity in subjects with LLD.

The primary outcome measure „change in GDS from baseline to end-of-treatment (week 10)“ is evaluated by a mixed model for repeated measures (MMRM) with fixed effects baseline, therapist, group, time and the interaction group*time (ARH1-structured covariance matrix over time) with corresponding marginal means and contrast tests. Data from low recruiting therapists (i.e. < 4 subjects) are pooled by study centre. (In a supplementary analysis all therapists are pooled by centre.) The potential clustering of observations of participants by therapist or centre will be investigated by multilevel modelling. Since mixed models can be expected to yield valid results only in case of missingness-at-random, multiple imputation approaches are taken to assess the robustness of the results. Specifically, missing values due to death, illness or chance are separately imputed assuming mixtures of missingness-not-atrandom patterns. Imputation data sets are post-processed by multiplication with factors and addition of offsets (tipping point analysis). Proxy measures are taken into account to ameliorate the effects of attrition.

### 2.6.4 Secondary analyses

The secondary objective of this study is to test the efficacy of LLD-CBT in comparison with SUI on patient reported outcome in major depressive disorder, anxiety, sleep, cognition, quality of life and overall health status. Furthermore, we will be investigating the influence of childhood traumatic experiences and personality traits on the change of depressive symptoms in LLDCBT in comparison to SUI.

Secondary outcomes (i.e. further time points and measures) are analyzed along the same lines, i.e. using mixed modeling (or GEEs). Time-to-event (e.g. drop-out or survival) distributions are summarized by the

Kaplan-Meier method and compared by the (stratified) log-rank test. Analysis of the set of subjects essentially observed and treated per protocol (PP) is supportive; similarly the compliers' average causal effect (CACE) is calculated [11]. Subgroup analyses are done by study site and gender (expected male to female ratio 1:2); interaction with treatment is investigated. Quantitative outcomes are summarized by mean, standard deviation and percentiles (i.e. 0, 25, 50, 75, 100), qualitative outcomes by count (percentage).

#### 2.6.4.1 Safety/Tolerability

All safety and tolerability parameters (adverse events, serious adverse events) will be listed by subject and treatment group. All analyses will be performed for the safety population and stratified by treatment group.

##### 2.6.4.1.1 Adverse events

Adverse events are listed and summarized by group and category, seriousness, severity, relatedness, respectively.

##### 2.6.4.1.2 Laboratory parameters

The study physician will take 4 tubes of venous blood (max. 50 ml in total per visit) from the subject at baseline, treatment, end-of-treatment and follow-up. The blood analyses include:

- Genetic and epigenetic analyses
- Measurement of Amyloid-β and Neurofilament light chain (NFL)
- miRNA analyses
- Metabolomics and Proteomics
- PBMC analyses

A Magnetic Resonance Imaging (MRI) acquisition of the subjects' brain will be performed at baseline, end-of-treatment and follow-up to obtain a high-resolution structural image and a resting-state fMRI of the subjects' brain before the psychotherapeutic intervention, at end-oftreatment and at follow-up. It will be complemented by Diffusion Tensor Imaging (DTI) of the subjects' brain.

Descriptive statistical analyses include: number of subjects (n), arithmetic mean, standard deviation (SD), median, first quartile (Q1), third quartile (Q3), minimum (min), maximum (max) for quantitative variables and absolute and relative frequencies for qualitative variables.

#### 2.6.4.2 Quality of life

Quality of life is assessed with WHO Quality of Life (WHOQOL-OLD, WHOQOL-BREF). Descriptive statistical analyses include: number of subjects (n), arithmetic mean, standard deviation (SD), median, first quartile (Q1), third quartile (Q3), minimum (min), maximum (max).

### 2.6.5 Planned subgroup analyses

Subgroup analyses are done by study site, gender (expected male to female ratio 1:2) and disease severity (moderate, severe).

#### 2.6.6 Interim analyses

There is no interim analysis planned.

### 2.7 Data problems

Not expected. If any, circumstances and treatment will be documented in the final analysis report.

### 2.8 Software

Data preparation and statistical analyses will be performed using SPSS Statistics (IBM Corp., Armonk, NY, USA). Other software may be used if required.

#### 2.8.1 List of abbreviations

| Abbreviation | Term |
|---|---|
| AE | Adverse Event |
| BMI | Body Mass Index |
| CACE | compliers' average causal effect |
| CBT | cognitive behaviour therapy |
| FAS | Full analysis set |
| GDS | Geriatric Depression Scale |
| LLD | Late life depression |
| PP | Per-protocol |
| PPS | Per-protocol-set |
| PRN | Pro re nata / as the situation demands; whenever needed |
| SAE | Serious Adverse Events |
| SAF | Safety analysis set |
| SUI | supportive unspecific intervention |
| WHO | World Health Organization |
