# Supplementary material for: Perceived Physical Health and Cognitive Behavioral Therapy vs Supportive Psychotherapy Outcomes in Adults With Late-Life Depression: A Secondary Analysis of a Randomized Clinical Trial
Source: JAMA Netw Open. 2024 Apr 15;7(4):e245841. doi: 10.1001/jamanetworkopen.2024.5841 (PMC11019392; doi:10.1001/jamanetworkopen.2024.5841)
Supplement: Supplement 2. — eFigure. CONSORT Diagram eTable 1. Comparison of Outcome Measures Between the LLD-CBT and SUI Group (ITT Population) Over the Course of the Treatment eTable 2. Model Summary of the Hierarchical Logistic Regression Analyses of Response and Remission at the End-of-Treatment and Follow-Up on the Variables of Interest eTable 3. Results of the Hierarchical Logistic Regression Analyses of Response and Remission at the End-of-Treatment and Follow-Up for All Variables of Interest [file jamanetwopen-e245841-s002.pdf]

Supplementary Online Content

Dafsari FS, Bewernick B, Böhringer S, et al. Perceived physical health and cognitive behavioral therapy vs supportive psychotherapy outcomes in adults with late-life depression: a secondary analysis of a randomized clinical trial. *JAMA Netw Open*. 2024;7(4):e245841. doi:10.1001/jamanetworkopen.2024.5841

eFigure. CONSORT Diagram

eTable 1. Comparison of Outcome Measures Between the LLD-CBT and SUI Group (ITT Population) Over the Course of the Treatment

eTable 2. Model Summary of the Hierarchical Logistic Regression Analyses of Response and Remission at the End-of-Treatment and Follow-Up on the Variables of Interest

eTable 3. Results of the Hierarchical Logistic Regression Analyses of Response and Remission at the End of Treatment and Follow-Up for All Variables of Interest

This supplementary material has been provided by the authors to give readers additional information about their work.

© 2024 Dafsari FS et al. *JAMA Network Open.*

eFigure 1. CONSORT Diagram. Source: Dafsari FS et al. Cognitive Behavioral Therapy for Late-Life Depression (CBTlate): Results of a Multicenter, Randomized, Observer-Blinded, Controlled Trial. Psychother Psychosom. 2023;92(3):180-192.

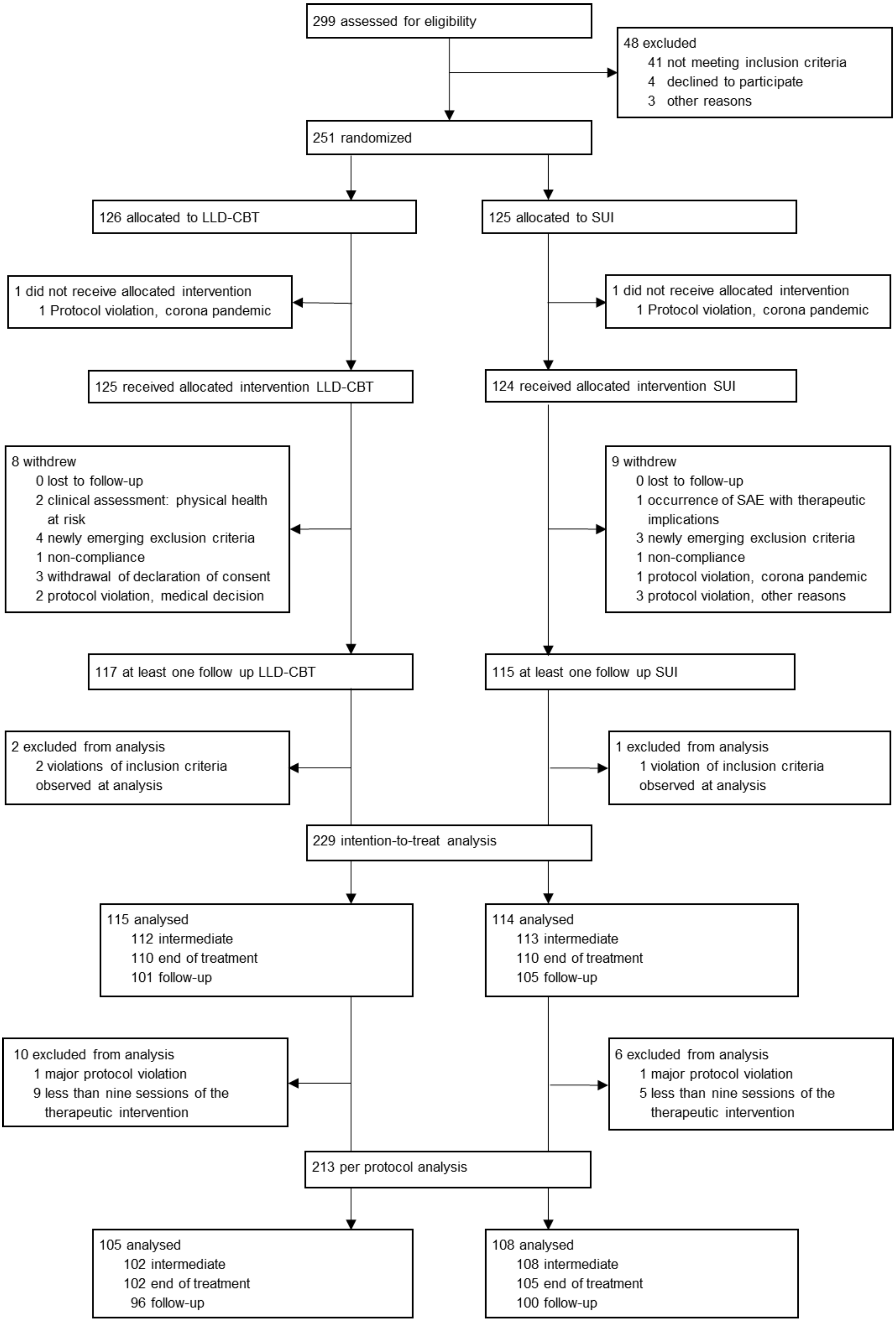

© 2024 Dafsari FS et al. *JAMA Network Open.*

eTable 1. Comparison of outcome measures between the LLD-CBT and SUI group (ITT population) over the course of the treatment. CBT = cognitive behavioral therapy. GDS = Geriatric Depression Scale. SUI = supportive unspecific intervention. WHOQOL-BREF = WHO Quality of Life Bref.

	**LLD-CBT**		**SUI**		**Overall**	
	**No.**	**Score, Mean (SD)**	**No.**	**Score, Mean (SD)**	**No.**	**Score, Mean (SD)**
**GDS score (possible range 0-30)**						
Baseline	115	21.0 (4.3)	114	20.4 (4.2)	229	20.7 (4.3)
Intermediate (week 5)	112	15.6 (6.6)	113	16.9 (5.4)	225	16.3 (6.0)
End of treatment (week 10)	109	13.5 (7.6)	110	14.2 (6.9)	219	13.8 (7.3)
Follow-up (month 6)	101	14.0 (7.4)	105	14.9 (6.6)	206	14.5 (7.0)
**WHOQOL-BREF physical health subscale score (possible range 0-100)**						
Baseline	112	53.8 (15.8)	112	52.5 (14.5)	224	53.1 (15.1)
Intermediate (week 5)	107	60.0 (16.4)	111	57.0 (15.7)	218	58.5 (16.0)
End of treatment (week 10)	106	62.5 (17.5)	106	59.7 (17.3)	212	61.1 (17.4)
Follow-up (month 6)	97	62.0 (17.9)	97	61.5 (17.4)	194	61.8 (17.6)

eTable 2. Model summary of the hierarchical logistic regression analyses of response and remission at the end-of-treatment and follow-up on the variables of interest.

	χ^2	df	P value	R^2
**Response at end-of treatment (N=197)**				
Model 1	11.872	3	**.008****	.079
Model 2	13.023	5	**.023***	.086
Model 3	20.810	7	**.004****	.135
**Response at follow-up (N=184)**				
Model 1	4.420	3	.220	.033
Model 2	5.562	5	.351	.041
Model 3	18.796	7	**.009****	.135
**Remission at end-of-treatment (N=197)**				
Model 1	19.022	3	**.001****	.126
Model 2	19.750	5	**.001****	.131
Model 3	31.176	7	**<.001****	.201
**Remission at follow-up (N=184)**				
Model 1	4.933	3	.177	.037
Model 2	5.555	5	.352	.042
Model 3	22.591	7	**.002****	.163

Model 1 includes predictors: age, gender, baseline GDS score. Model 2 includes the additional predictors: number of physical diseases at baseline, Charlson Comorbidity Index at baseline. Model 3 includes all predictors of model 1 and 2 and the additional predictors: perceived physical health score (WHOQOL-BREF subscale) at baseline, Interaction perceived physical health score*treatment. *p < .05. **p < .01. df = degree of freedom.

© 2024 Dafsari FS et al. *JAMA Network Open.*

eTable 3. Results of the hierarchical logistic regression analyses of response and remission at the end of treatment and follow-up for all variables of interest.

Variables	B	SE	Wald	df	P value	Odds Ratio	95% CI lower	95% CI upper
**Response at end of treatment**								
**Model 1**								
Constant	5.503	1.850	8.845	1	**.003****	245.42		
Age	-.064	.023	8.089	1	**.004****	.94	.897	.980
Gender	.338	.321	1.105	1	.293	1.40	.747	2.633
Baseline GDS score	-.079	.036	4.775	1	**.029***	.92	.861	.992
**Model 2**								
Constant	5.457	1.913	8.134	1	**.004****	234.42		
Age	-.066	.025	6.711	1	**.010***	.94	.891	.984
Gender	.332	.323	1.058	1	.304	1.39	.740	2.624
Baseline GDS score	-.081	.036	4.905	1	**.027***	.92	.859	.991
Number of physical diseases at baseline	.098	.092	1.130	1	.288	1.10	.921	1.321
Charlson Comorbidity Index (CCI) at baseline	-.043	.115	.140	1	.708	.96	.764	1.200
**Model 3**								
Constant	3.846	2.042	3.549	1	**.060***	46.82		
Age	-.071	.026	7.440	1	**.006****	.93	.885	.980
Gender	.239	.333	.515	1	.473	1.27	.661	2.438
Baseline GDS score	-.061	.038	2.560	1	.110	.94	.873	1.014
Number of physical diseases at baseline	.141	.096	2.139	1	.144	1.15	.953	1.390
Charlson Comorbidity Index (CCI) at baseline	-.030	.118	.066	1	.797	.97	.770	1.222
Perceived physical health score (WHOQOL-BREF) at baseline	.036	.014	6.857	1	**.009****	1.04	1.009	1.065
Interaction perceived physical health*treatment	-.005	.006	.930	1	.335	.99	.984	1.006
**Response at follow-up**								
**Model 1**								
Constant	2.868	1.923	2.223	1	.136	17.60		
Age	-.048	.024	3.985	1	**.046***	.95	.909	.999
Gender	-.005	.339	.000	1	.988	.99	.512	1.934
Baseline GDS score	-.011	.039	.075	1	.785	.99	.917	1.067
**Model 2**								
Constant	2.429	1.981	1.503	1	.220	11.35		
Age	-.039	.027	2.144	1	.143	.96	.912	1.013
Gender	-.016	.340	.002	1	.962	.98	.505	1.916
Baseline GDS score	-.010	.039	.067	1	.796	.99	.918	1.068
Number of physical diseases at baseline	.076	.096	.620	1	.431	1.08	.893	1.303
Charlson Comorbidity Index (CCI) at baseline	-.124	.130	.903	1	.342	.88	.685	1.140
**Model 3**								
Constant	-.030	2.195	.000	1	.989	.97		
Age	-.048	.029	2.884	1	.089	.95	.901	1.007
Gender	-.165	.359	.212	1	.645	.85	.419	1.713
Baseline GDS score	.027	.042	.395	1	.530	1.03	.945	1.116
Number of physical diseases at baseline	.139	.102	1.836	1	.175	1.15	.940	1.404
Charlson Comorbidity Index (CCI) at baseline	-.114	.138	.680	1	.409	.89	.681	1.170
Perceived physical health score (WHOQOL-BREF) at baseline	.051	.015	11.235	1	**<.001****	1.05	1.021	1.085
Interaction perceived physical health*treatment	-.007	.006	1.392	1	.238	.99	.982	1.005
**Remission at end of treatment**								
**Model 1**								

© 2024 Dafsari FS et al. *JAMA Network Open.*

Constant	6.896	1.987	12.041	1	**<.001****	988.46		
Age	-.069	0.024	8.343	1	**.004****	0.93	0.890	0.978
Gender	0.153	0.331	0.215	1	.643	1.17	0.610	2.229
Baseline GDS score	-.135	0.039	12.184	1	**<.001****	0.87	0.810	0.943
**Model 2**								
Constant	7.039	2.055	11.732	1	**<.001****	1140.40		
Age	-.075	0.027	7.771	1	**0.005****	0.93	0.881	0.978
Gender	0.149	0.332	0201	1	.654	1.16	0.606	2.224
Baseline GDS score	-.136	0.039	12.328	1	**<.001****	0.87	0.809	0.942
Number of physical diseases at baseline	0.069	0.096	0.521	1	.470	1.07	0.888	1.293
Charlson Comorbidity Index (CCI) at baseline	0.010	0.117	0.008	1	.930	1.01	0.803	1.272
**Model 3**								
Constant	5.161	2.189	5.560	1	**.018***	174.32		
Age	-.084	0.028	9.085	1	**.003****	0.92	0.871	0.971
Gender	0.023	0.346	0.004	1	.948	1.02	0.519	2.016
Baseline GDS score	-.116	0.041	8.043	1	**.005****	0.89	0.822	0.965
Number of physical diseases at baseline	0.119	0.101	1.402	1	.236	1.13	0.925	1.372
Charlson Comorbidity Index (CCI) at baseline	0.037	0.121	0.094	1	.760	1.04	0.819	1.314
Perceived physical health score (WHOQOL-BREF) at baseline	0.044	0.015	9.360	1	**.002****	1.05	1.016	1.076
Interaction perceived physical health*treatment	-.005	0.006	0.844	1	.358	0.99	0.983	1.006
**Remission at follow-up**								
**Model 1**								
Constant	3.257	1.970	2.732	1	.098	25.97		
Age	-.044	.025	3.185	1	.074	.96	.912	1.004
Gender	-.172	.342	.253	1	.615	.84	.431	1.646
Baseline GDS score	-.043	.039	1.223	1	.269	.96	.887	1.034
**Model 2**								
Constant	2.910	2.030	2.056	1	.152	18.36		
Age	-.037	.027	1.824	1	.177	.96	.913	1.017
Gender	-.180	.343	.275	1	.600	.84	.427	1.636
Baseline GDS score	-.043	.039	1.188	1	.276	.96	.887	1.035
Number physical diseases at baseline	.055	.098	.311	1	.577	1.06	.872	1.280
Charlson Comorbidity Index (CCI) at baseline	-.094	.131	.519	1	.471	.91	.705	1.176
**Model 3**								
Constant	.289	2.262	.016	1	.898	1.34		
Age	-.048	.029	2.667	1	.102	.95	.900	1.010
Gender	-.386	.368	1.101	1	.294	.68	.330	1.398
Baseline GDS score	-.007	.043	.023	1	.879	.99	.913	1.081
Number physical diseases at baseline	.120	.105	1.316	1	.251	1.13	.918	1.385
Charlson Comorbidity Index (CCI) at baseline	-.077	.140	.304	1	.582	.93	.703	1.219
Perceived physical health score (WHOQOL-BREF) at baseline	.061	.016	14.471	1	**<.001****	1.06	1.030	1.096
Interaction perceived physical health*treatment	-.010	.006	2.498	1	.114	.99	.979	1.002

* $p < .05$. ** $p < .01$. df = degree of freedom. CCI = Charlson Comorbidity Index. WHOQOL-BREF = WHO Quality of Life Bref questionnaire.

© 2024 Dafsari FS et al. *JAMA Network Open.*
