# Supplementary material for: Perceived Physical Health and Cognitive Behavioral Therapy vs Supportive Psychotherapy Outcomes in Adults With Late-Life Depression: A Secondary Analysis of a Randomized Clinical Trial
Source: JAMA Netw Open. 2024 Apr 15;7(4):e245841. doi: 10.1001/jamanetworkopen.2024.5841 (PMC11019392; doi:10.1001/jamanetworkopen.2024.5841)
Supplement: Supplement 3. — Data Sharing Statement [file jamanetwopen-e245841-s003.pdf]

Data Sharing Statement

Dafsari. Perceived Physical Health and Cognitive Behavioral Therapy vs Supportive Psychotherapy Outcomes in Adults With Late-Life Depression. *JAMA Netw Open*. Published April 15, 2024. doi:10.1001/jamanetworkopen.2024.5841

Data
Data available: Yes
Data types: Deidentified participant data, Data dictionary, Other (please specify)
Additional Information: The full study protocol and the statistical analysis plan are available in the supplement of the publication. The data that support the findings of this study are not publicly available due to ethics restrictions but available to investigators from the corresponding author upon reasonable request with individual permission from the review committee and the local institutions ethics board. Researchers who provide a methodologically sound proposal should direct these to forugh.salimi-dafsari@uk-koeln.de and data requestors will need to sign a data access agreement with the study sponsor (University of Cologne, Germany).
How to access data: The full study protocol and the statistical analysis plan are available in the supplement of the publication. The data that support the findings of this study are not publicly available due to ethics restrictions but available to investigators from the corresponding author upon reasonable request with individual permission from the review committee and the local institutions ethics board. Researchers who provide a methodologically sound proposal should direct these to forugh.salimi-dafsari@uk-koeln.de and data requestors will need to sign a data access agreement with the study sponsor (University of Cologne, Germany).
When available: With publication

Supporting Documents
Document types: None

Additional Information
Who can access the data: The full study protocol and the statistical analysis plan are available in the supplement of the publication. The data that support the findings of this study are not publicly available due to ethics restrictions but available to investigators from the corresponding author upon reasonable request with individual permission from the review committee and the local institutions ethics board. Researchers who provide a methodologically sound proposal should direct these to forugh.salimi-dafsari@uk-koeln.de and data requestors will need to sign a data access agreement with the study sponsor (University of Cologne, Germany).
Types of analyses: The full study protocol and the statistical analysis plan are available in the supplement of the publication. The data that support the findings of this study are not publicly available due to ethics restrictions but available to investigators from the corresponding author upon reasonable request with individual permission from the review committee and the local institutions ethics board. Researchers who provide a methodologically sound proposal should direct these to forugh.salimi-dafsari@uk-koeln.de and data requestors will need to sign a data access agreement with the study sponsor (University of Cologne, Germany).
Mechanisms of data availability: The full study protocol and the statistical analysis plan are available in the supplement of the publication. The data that support the findings of this study are not publicly available due to ethics restrictions but available to investigators from the corresponding author upon reasonable request with individual permission from the review committee and the local institutions ethics board. Researchers who provide a methodologically sound proposal should direct these to forugh.salimi-dafsari@uk-koeln.de and data requestors will need to sign a data access agreement with the study sponsor (University of Cologne, Germany).
